# Targeting DDX3X suppresses progression of KRAS-driven lung cancer by disrupting antioxidative homeostasis and inducing ferroptosis

**DOI:** 10.1038/s41419-025-07980-8

**Published:** 2025-08-30

**Authors:** Meijuan Dian, Liang Yun, Qingyu Meng, Songwen Lin, Ming Ji, Ying Zhou, Wenqian Liu, Zhuoying Yang, Yayan Zhao, Gaoyuan Li, Jianjun Jiang, Weichao Hao, Zhijie Chen, Zehao Zhou, Ruihao Zhang, Tianyuan Liu, Yujing He, Tianbao Yan, Haofei Wang, Shane J. F. Cronin, Josef M. Penninger, Kaican Cai, Shuan Rao

**Affiliations:** 1 Department of Thoracic Surgery, Nanfang Hospital, Southern Medical University, Guangzhou, China; 2 Department of Radiation Oncology, Peking Union Medical College Hospital, Chinese Academy of Medical Sciences & Peking Union Medical College, Beijing, China; 3 State Key laboratory of Bioactive Substances and Functions of Natural Medicines, Institute of Materia Medica, Chinese Academy of Medical Sciences & Peking Union Medical College, Beijing, China; 4 Cancer Research Institute, School of Basic Medical Sciences, Southern Medical University, Guangzhou, China; 5 Department of Oncology, The First Affiliated Hospital of Guangdong Pharmaceutical University, Guangzhou, China; 6 State Key Laboratory of Medical Molecular Biology, Department of Biochemistry and Molecular Biology, Haihe Laboratory of Cell Ecosystem, Institute of Basic Medical Sciences, Chinese Academy of Medical Sciences, School of Basic Medicine, Peking Union Medical College, Beijing, China; 7 Tsukuba Life Science Innovation Program, University of Tsukuba, Tsukuba, Japan; 8 Department of Laboratory Medicine, Medical University of Vienna, Vienna, Austria; 9 Institute of Molecular Biotechnology of the Austrian Academy of Sciences, Vienna, Austria; 10 Department of Medical Genetics, Life Sciences Institute, University of British Columbia, Vancouver, British Columbia Canada; 11 Helmholtz Centre for Infection Research, Braunschweig, Germany

**Keywords:** Cancer, Drug development

## Abstract

Approximately 30% of human cancers carry various *RAS* mutations, including *KRAS*, *NRAS*, and *HRAS*. Among these mutations, *KRAS* is the most prevalent isoform detected in lung cancer. While several small molecular inhibitors targeting specifically *KRAS*^*G12C*^ have been developed and tested clinically, alternative approaches are still necessary due to expected drug resistance. In this study, we present evidence that the loss of DDX3X significantly delays tumor progression in various *KRAS*-driven lung cancer models. Inhibition of DDX3X disrupts cysteine and glutathione metabolism, thereby inducing ferroptosis in lung cancer cells. This effect is primarily mediated by the downregulation of Cystathionine-β-synthase (CBS), the rate-limiting enzyme in cysteine generation. Mechanistically, DDX3X directly binds to the transcription factor *JUND*, which mediates the transcriptional regulation of METTL16, a key N^6^-methyladenosine methyltransferase, and subsequently regulates m^6^A modification and translation of *CBS* transcripts. This cascade induces hypermethylation and high expression of CBS, consequently triggering cysteine production and maintaining antioxidative homeostasis, which is essential for the survival of *KRAS*-driven lung cancer cells. Finally, we demonstrate that a newly developed DDX3X PROTAC degrader J10 efficiently delays lung cancer progression with multiple advantages compared to DDX3X small molecular inhibitor RK-33 and limited side effects. These findings unveil the potential of DDX3X as a valuable target for adjuvant therapies in managing *KRAS*-driven lung cancer.

## Introduction

Lung cancer stands as the leading cause of cancer-related fatalities worldwide, with non-small cell lung cancer (NSCLC) accounting for approximately 85% of all cases [1]. NSCLC frequently arises from oncogenic mutations, including *EGFR*, *KRAS*, *ALK*, *MET* and so on [2]. For a considerable time, KRAS has been perceived as an elusive target for drug development. However, recent advances have led to the creation and clinical testing of small molecular inhibitors specifically targeting KRAS^G12C^ [3]. Despite these strides, it is indicated that lung cancer cells can swiftly develop resistance to these treatments [4]. Consequently, there remains an urgent and pressing need to decipher the molecular mechanisms driving *KRAS*-driven lung cancer progression and to explore alternative therapeutic strategies.

DDX3X is an RNA-binding protein classified within the DEAD-box helicase family. It serves diverse functions in RNA homeostasis, encompassing transcriptional regulation [5], splicing [6], mRNA stability [7], mRNA nuclear export [8], ribosomal biogenesis [9], and translation [10]. Accumulating evidence underscores DDX3X involvement in various cellular processes, including cell-cycle regulation [11], innate immune response [12], apoptosis [13], DNA damage [14], embryogenesis [15] and stress response [16]. Whether DDX3X is involved in cellular metabolic homeostasis remains poorly understood. While genetic alterations in DDX3X have been identified in several cancers, its role in cancer progression remains a subject of debate and varies depending on the cancer context. For instance, DDX3X acts as an oncogenic gene in pancreatic cancer, regulating either transcription or translation pathways to promote tumor progression [17]. DDX3X enhances tumor invasion or cell growth in colorectal cancer [18]. DDX3 regulates castration-resistant prostate cancer by mediating posttranscriptional regulation of the androgen receptor [19]. The loss of DDX3X perturbed lymphomagenesis in a sex-dependent manner [20]. However, recent studies have revealed that DDX3 might be a tumor suppressor in specific cancer contexts. In advanced colorectal cancer [21] and p53-inactivated lung tumors [22], diminished expression of DDX3 was observed, correlating with poor prognosis in patients, while in vitro experiments, loss of DDX3 promoted soft-agar growth and invasion. These observations highlight the important and multiple roles of DDX3X in cancer progression.

In lung cancer, high expression of DDX3X has been associated with more aggressive tumor behavior. Inhibition of DDX3X disrupts the DDX3X-β-catenin axis and the non-homologous end joining (NHEJ) DNA damage repair pathway [23]. A small molecule DDX3X inhibitor, RK-33, has demonstrated significant potential in promoting tumor regression and enhancing sensitivity to radiotherapy [23]. In our study, by utilizing a spontaneous *KRAS*-driven lung cancer model, human lung cancer organoids, and cell lines, we have unveiled a pivotal role of DDX3X in lung cancer progression. Specifically, inhibiting DDX3X disrupts cysteine and glutathione anabolism, consequently inducing ferroptosis in lung cancer cells. This effect is primarily mediated by the suppression of CBS translation through JUND/METTL16-mediated m^6^A modification. Additionally, we have developed novel DDX3X PROTAC degraders and compared their anti-tumor efficiency with RK-33, showcasing their great potential and advantages in targeting DDX3X for lung cancer therapeutics. In conclusion, our findings elucidate the critical function of DDX3X in maintaining antioxidant homeostasis and provide an alternative strategy to treat *KRAS*-driven lung cancer.

## Results

### Disrupting *Ddx3x* impedes lung cancer progression and markedly extends survival in mice harboring *Kras*^*G12D*^-driven lung cancer

**Fig. 1:**
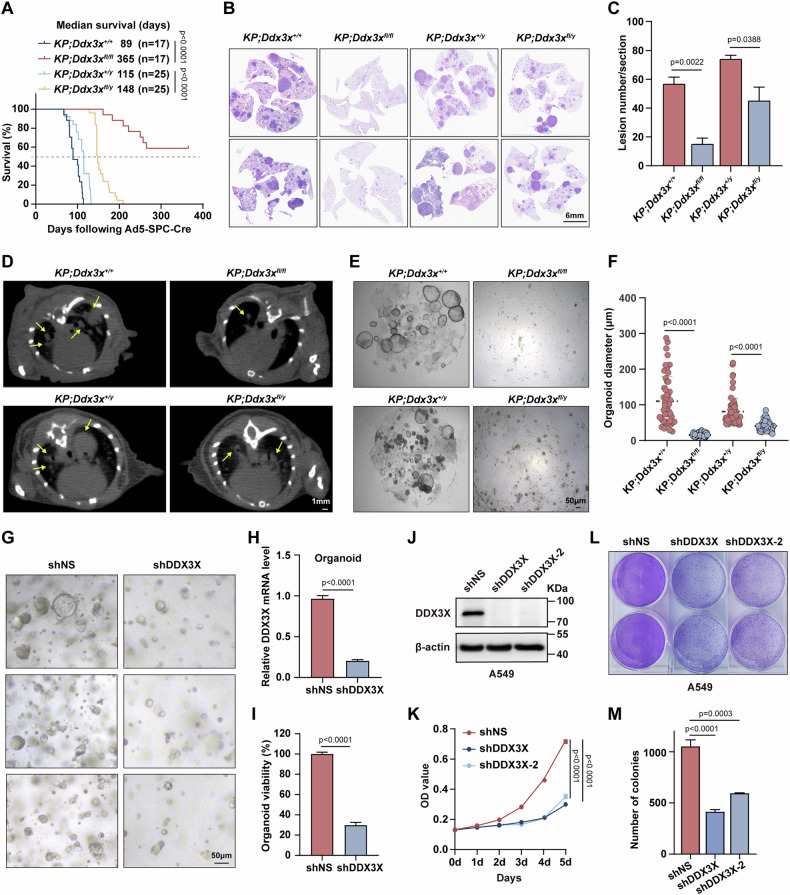
Depletion of *Ddx3x* delays lung cancer progression and markedly extends survival in mice harboring *Kras*^*G12D*^-driven lung cancer. **A** Kaplan-Meier analysis of the overall survival of mice bearing lung cancer: *KP;Ddx3x*^*+/+*^ (n = 17), *KP;Ddx3x*^*fl/fl*^ (n = 17), *KP;Ddx3x*^*+/y*^ (n = 25), and *KP;Ddx3x*^*fl/y*^ (n = 25). (Log-rank (Mantel-Cox) test). **B** Representative hematoxylin and eosin (H&E) stained lung tissue sections from 12-week-old mice of *KP;Ddx3x*^*+/+*^, *KP;Ddx3x*^*fl/fl*^, *KP;Ddx3x*^*+/y*^, and *KP;Ddx3x*^*fl/y*^ mice; Scale bar: 6 mm. **C** Quantification of lesion numbers in **(B)** (mean ± SEM, unpaired Student’s t test). **D** Representative transverse micro-CT images of wild-type (WT) and DDX3X knockout (KO) mice were captured at uniform endpoint event time points. The selected images correspond to the location of the largest tumour diameter. Tumors are marked with yellow arrows; Scale bar: 1 mm. **E** Representative bright-field microscopy images of lung cancer organoids captured on day 6 of culture, derived from mice with genotypes: *KP;Ddx3x*^*+/+*^, *KP;Ddx3x*^*fl/fl*^, *KP;Ddx3x*^*+/y*^, and *KP;Ddx3x*^*fl/y*^; Scale bar: 50 μm. **F** Quantification of the diameters of organoids from **(E)** (mean ± SEM, One-way ANOVA). **G** Representative bright-field images of control and DDX3X knockdown patient-derived lung cancer organoids. Scale bar: 50 μm. **H** Analysis of DDX3X mRNA levels in patient-derived organoids following lentiviral transduction with non-targeting shRNA (shNS) or shRNA targeting DDX3X (shDDX3X) (*n* = 3, mean ± SEM, unpaired Student’s t test). **I** Quantification of the viability of patient-derived lung cancer organoids using an ATP release assay (CellTiter-Glo 3D) (*n* = 4, mean ± SEM, unpaired Student’s t test). **J** Immunoblot analysis of DDX3X expression in A549 cells transduced with shNS or shDDX3X. *β*-actin was employed as a loading control. **K** A 5-day period analysis of the cell proliferation in shNS or shDDX3X A549 cells (*n* = 3, mean ± SEM, Two-way ANOVA). **L** Clonogenic assay of A549 cells transduced with shNS or shDDX3X, the colonies were stained and assessed after a 2-week incubation period. **M** Quantification of colony formation in A549 cells transduced with shNS or shDDX3X. (*n* = 3, mean ± SEM, unpaired Student’s *t* test).

To investigate the correlation between DDX3X expression and lung cancer patient prognosis, we conducted a comprehensive analysis of survival data from lung cancer cases profiled by TCGA. Notably, elevated DDX3X expression was associated with poorer overall survival and progression-free intervals in lung adenocarcinoma (LUAD) (Fig. S1A, B). To explore the role of DDX3X in lung cancer, we initially bred *Ddx3x* ^*fl/ fl*^ mice to lox-stop-lox-*Kras*^*G12D*^ mice to generate *Kras*^*G12D*^*;Ddx3x*^*fl/fl*^ and *Kras*^*G12D*^*;Ddx3x*^*fl/y*^ offspring. Lung cancer was induced in a stepwise process via intratracheal administration of Ad5-Spc-Cre virus [24]. Notably, we observed no lung cancer related deaths in *Kras*^*G12D*^*;Ddx3x*^*fl/fl*^ and only a limited number in *Kras*^*G12D*^;*Ddx3x*^*fl/y*^ mice (two out of 12), underscoring a remarkable survival advantage compare to their littermates (Fig. S1C). Subsequently, by introducing *p53*^*fl/fl*^ allele, we obtained *Kras*^*G12D*^*;Ddx3x*^*fl/fl*^ *p53*^*fl/fl*^ (termed *KP*;*Ddx3x*^*fl/fl*^ hereafter), *KP*;*Ddx3x*^*fl/+*^ and *KP*;*Ddx3x*^*fl/y*^ mice, similarly, our data showed that depletion of *Ddx3x* also prolonged survival of mice bearing lung cancer dramatically (Fig. 1A), such survival benefit was also evident in *KP*;*Ddx3x*^*fl/+*^ mice when only a single *Ddx3x* gene copy was eliminated (Fig. S1D). Furthermore, histological evaluation 12 weeks after tumor induction demonstrated much less tumor burden and fewer tumor foci in *KP*;*Ddx3x*^*fl/fl*^ and *KP*;*Ddx3x*^fl/y^ mice compared to their DDX3X competent littermates (Fig. 1B, C). Consistently, micro-CT analysis also demonstrated that deletion of DDX3X significantly delayed lung tumor progression (Fig. 1D and Fig. S1E). Next, we purified primary lung tumor cells and conducted tumor spheroid assays, which indicated that ablation of DDX3X significantly impaired tumor spheroid formation (Fig. 1E, F). Similarly, in lung cancer patient-derived organoids (PDOs), which faithfully reproduce the morphological and genomic features of human lung tumors (Fig. S2), we observed that both the size and viability were markedly decreased in DDX3X deficient lung tumor organoids (Fig. 1G–I). Moreover, we generated DDX3X-knock down cells in a KRAS mutant human lung cancer cell A549 (KRAS^G12S^) and found suppression of DDX3X halted the proliferation of A549 cells (Fig. 1J–M). A similar phenotype was reproduced in PC-9 cells (KRAS wildtype) and NCI-H23 cells (KRAS^G12C^) (Fig. S1F–M).

### DDX3X is a pivotal regulator of cysteine-glutathione metabolism and ferroptosis in lung cancer cells

**Fig. 2:**
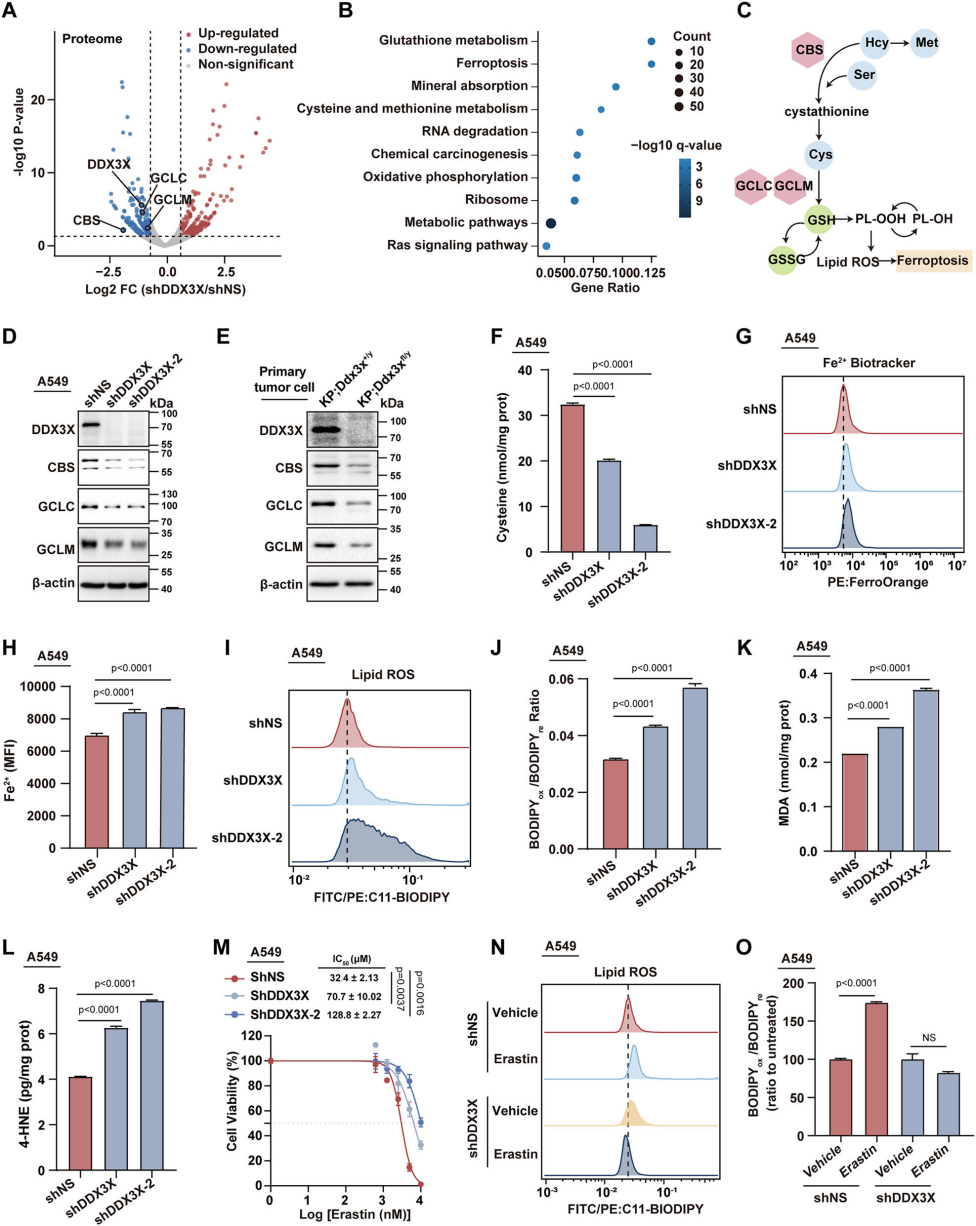
DDX3X controls cysteine-glutathione metabolism and ferroptosis in lung cancer cells. **A** Volcano plot illustrates the fold change (FC) and variance for all expressed proteins in shNS or shDDX3X A549 cells. Differentially expressed proteins (DEP) are denoted by red and blue points, indicating up-regulated (fold change>1.5 and *p*-Value < 0.05) and down-regulated (fold change<0.6 and *p*-Value < 0.05) changes, respectively. **B** Scatter plot shows the enrichment of top KEGG pathways based on differentially expressed proteins (DEP) from (**A)**. Point size and color are proportional to the DEP hit count (number shown) and q value, respectively. The top ten KEGG pathways are ranked by gene ratio. **C** A pathway map highlights proteins and biochemicals within the ferroptosis pathway, specifically highlighting Cysteine-Glutathione signature proteins as indicated in **(A)**. **D** Immunoblot analysis of DDX3X, CBS, GCLC, and GCLM in A549 cells. β-actin was employed as a loading control. **E** Immunoblot analysis of DDX3X, CBS, GCLC, and GCLM in the indicated cells. β-actin was employed as a loading control. **F** Quantification of cysteine levels in A549 shDDX3X or shNS cells. *n* = 3, mean ± SEM with ordinary One-way ANOVA. **G** Flow cytometry assay of ferrous ion (Fe^2+^) fluorescence in shNS or shDDX3X A549 cells. **H** Quantification of mean fluorescence intensity (MFI) in **(G)** (*n* = 3, mean ± SEM, One-way ANOVA). **I** Flow cytometry analysis assessed lipid peroxidation through C11-BODIPY fluorescence in shNS or shDDX3X A549 cells. FITC-BODIPY C11 and PE-BODIPY C11 represent oxidized (ox) and reduced (re) populations, respectively**. J** Quantification of the ratio of oxidized (ox) to reduction (re) fluorescence intensity in **(I)** (*n* = 3, mean ± SEM, One-way ANOVA). **K**, **L** Quantification of malondialdehyde (MDA) **(K)**, and 4-hydroxynonenal (4-HNE) **(L)** levels in A549 shDDX3X or shNS cells. *n* = 3, mean ± SEM with ordinary One-way ANOVA. **M** Cell viability assay of shNS or shDDX3X A549 cells treated with or without 40 μM Erastin for 48 h (*n* = 3, mean ± SEM, Two-way ANOVA). **N** Flow cytometry analysis of shNS or shDDX3X A549 cells in the presence or absence of 40 μM Erastin for 48 h to assess lipid peroxidation through C11-BODIPY fluorescence. **O** The relative ratio of oxidized (ox) to reduction (re) fluorescence intensity, normalized to the untreated condition in **(N)** (*n* = 3, mean ± SEM, NS. p > 0.05, Two-way ANOVA).

To study the role of DDX3X in lung cancer progression, we undertook a label-free proteomic analysis using DDX3X knockdown (shDDX3X) A549 cells. Our findings unveiled a significant alteration in 248 upregulated proteins and 192 downregulated proteins (Fig. 2A). Pathway enrichment analysis (PEA) highlighted the enrichment of proteins associated with glutathione metabolism, ferroptosis, and cysteine and methionine metabolism in DDX3X knockdown cells (Fig. 2B). Concurrently, we executed an untargeted metabolome analysis on the DDX3X knockdown A549 cells. Consistent with the proteomic findings, key metabolites linked to cysteine and glutathione including s-adenosylmethionine (SAM), glutathione (GSH), glutathione oxidized (GSSG), and γ-Glutamylcysteine (gamma-Glu-Cys) [25] were markedly reduced in shDDX3X cells (Fig. S3A). This was further corroborated through KEGG metabolic enrichment pathway analysis via MetaboAnalyst [26](Fig. S3B) and targeted liquid chromatography with tandem mass spectrometry (LC-MS/MS) (Fig. S3C, D). Integrating the proteomic and metabolic datasets, we discovered that enzymes pivotal to cysteine biosynthesis, such as cystathionine beta-synthase (CBS) [27], and key glutathione biosynthesis genes, including glutamate-cysteine ligase catalytic subunit (GCLC) [28] and Glutamate-cysteine ligase regulatory subunit (GCLM) [29], were key downstream targets downregulated upon DDX3X inhibition (Fig. 2C). This was further validated through immunoblotting assays that confirmed the downregulation of CBS, GCLC, and GCLM in shDDX3X A549 cells (Fig. 2D), NCI-H23 cells (Fig. S3E), and primary lung tumor cells from *KP;Ddx3x*^*fl/y*^ mice (Fig. 2E). Accordingly, we observed a significant decrease in cysteine levels, which is an antioxidant metabolite regulated by cystathionine β-synthase (CBS), in shDDX3X cells (Fig. 2F and Fig. S3F). Given that compromised cysteine and glutathione metabolism can elevate lipid peroxidation [30], and with ferroptosis-associated genes also being prominent in our proteomic analysis (Fig. 2B), we evaluated intracellular Fe^2+^ and lipid peroxidation levels in shDDX3X cells using Ferene-S and the lipid oxidation probe C11 BODIPY 581/591, respectively. Consistently, both parameters were elevated in DDX3X-deficient A549, PC-9 and NCI-H23 cells (Fig. 2G–J and Fig. S3G–L). Furthermore, the levels of malondialdehyde (MDA) and 4-hydroxynonenal (4-HNE), both markers of oxidative stress-induced lipid peroxidation and ferroptosis, were significantly elevated in shDDX3X cells (Fig. 2K, L, Fig. S3M, N), indicating that DDX3X ablation instigates ferroptotic cell death in lung cancer cells. Notably, when we treated lung cancer cells with erastin, a ferroptosis inducer known to inhibit system Xc-mediated cystine import [31], we observed enhanced resistance to erastin treatment in shDDX3X cells (Fig. 2M). This was attributed to diminished efficacy of erastin in elevating lipid peroxidation levels in DDX3X-deficient cells compared to their wild-type control cells (Fig. 2N, O), likely due to the inherent saturation of lipid peroxidation in the absence of DDX3X.

### CBS emerges as the pivotal downstream effector of DDX3X in orchestrating lung cancer progression

**Fig. 3:**
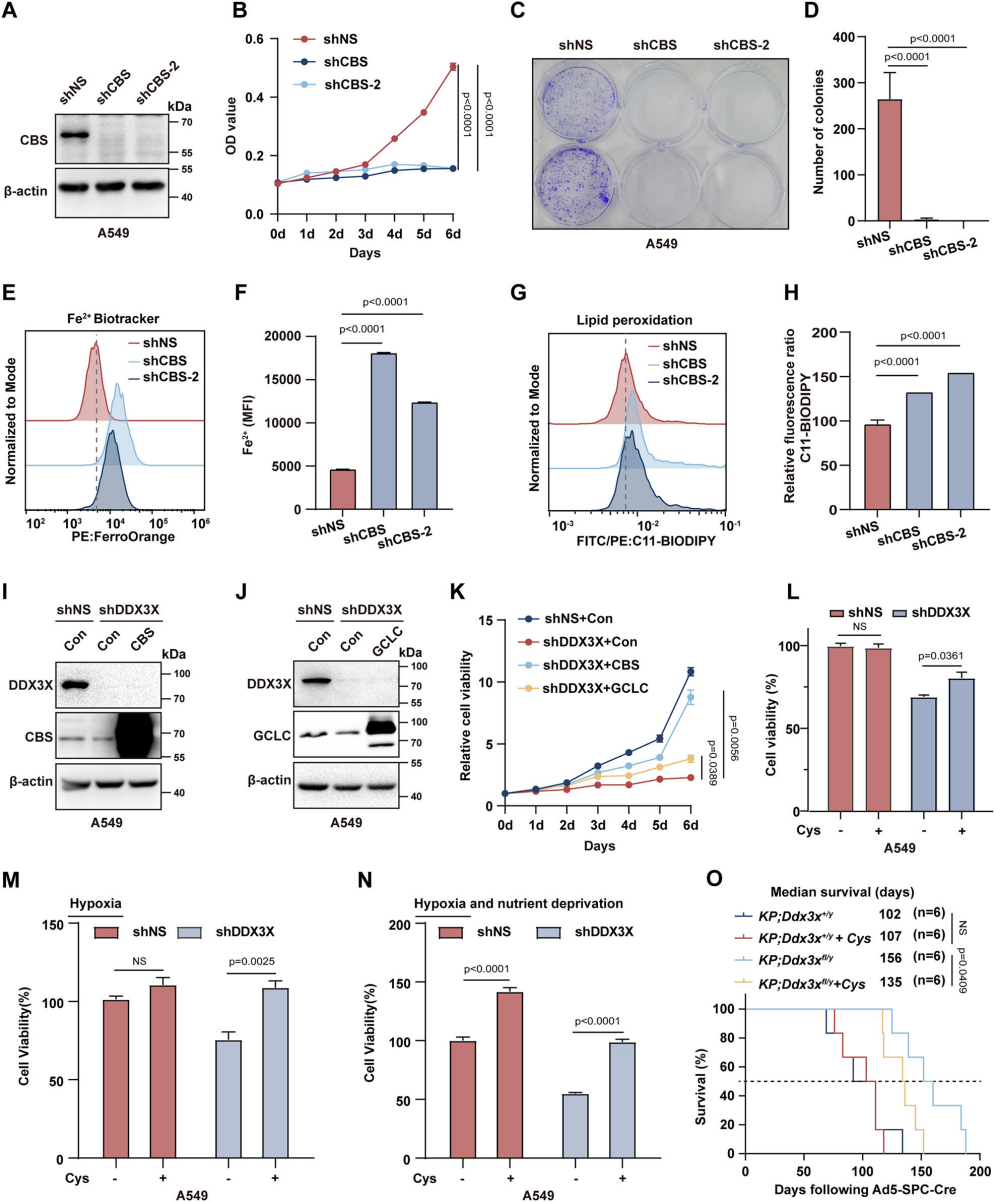
CBS is the key downstream effector of DDX3X in orchestrating lung cancer progression. **A** Representative western blot showing CBS-silenced A549 cells. β-actin was used as loading control. **B** Assessment of cell proliferation in CBS knockdown A549 cells using a CCK-8 assay for 6 days (*n* = 3, mean ± SEM, One-way ANOVA). **C** Clonogenic assay in control or CBS knockdown A549 cells. **D** Quantification of colony formation in **(C)**. **E** FACs analysis of ferrous ion (Fe^2+^) levels in CBS knockdown A549 cells. **F** Quantification of the mean fluorescence intensity (MFI) of ferrous ions (Fe^2+^) in **(E)** (*n* = 3, mean ± SEM, One-way ANOVA). **G** Assessment of lipid peroxidation via C11-BODIPY 581/591 staining in control or CBS knockdown A549 cells. **H** Quantification of C11-BODIPY fluorescence intensity in the specified cells in **(G)**, Data normalization was performed against measurements in shNS for comparative analysis (*n* = 3, mean ± SEM, One-way ANOVA). **I** Western blot analysis of DDX3X and CBS in A549 cells transduced with lentiviral expressing shNS, shDDX3X, shDDX3X plus overexpressed (OE) CBS. β-actin was used as loading control. **J** Western blot analysis of DDX3X and GCLC in A549 cells transduced with lentiviral shNS, shDDX3X, shDDX3X plus overexpressed GCLC. β-actin was used as loading control. **K** A 6-day assessment of relative cell viability for each indicated cell line. The shNS value was used for data normalization (*n* = 3, mean ± SEM, NS. *p* > 0.05, Two-way ANOVA). **L** Cell viability assay of control or DDX3X knockdown A549 cells treated with or without 0.5 mM cysteine for 24 hours. The untreated shNS-A549 cells was used for data normalization (*n* = 3, mean ± SEM, unpaired Student’s t test). **M**, **N** Cell viability analysis of DDX3X knockdown A549 cells with or without 0.5 mM cysteine supplementation for 24 h, under hypoxia alone **(M)** and in combination with nutrient deprivation **(N)**. **O** Kaplan-Meier survival curves depicting the overall survival of mice with lung cancer *(KP;Ddx3x*^*+/y*^ and *KP;Ddx3x*^*fl/y*^ *n* = 6), with mice subjected to either 1 mM cysteine-supplemented water or regular water (NS. p > 0.05, Log-rank (Mantel-Cox) test).

Given the downregulation of CBS, GCLC, and GCLM in DDX3X-deficient lung cancer cells, we then questioned which enzyme primarily mediates the effects of DDX3X inhibition. Since existing knowledge underscores the predominant role of GCLC over GCLM in modulating cancer progression [32, 33], we thereby individually silenced CBS and GCLC in lung cancer cells. Inhibition of CBS markedly suppressed proliferation in A549, PC-9 and NCI-H23 cells (Fig. 3A–D and Fig. S4A–D), accompanied by a notable rise in intracellular Fe^2+^ and lipid peroxidation levels (Fig. 3E–H and Fig. S4E–H). Notably, while knocking down GCLC yielded a similar phenotype, its impact was comparatively milder than what was observed in CBS suppression (Fig. S4I–T). Conversely, the overexpression of either CBS or GCLC in A549 cells led to accelerated cell proliferation and diminished Fe^2+^ and lipid peroxidation levels (Fig. S5A–H), underscoring the vital roles of these enzymes in modulating antioxidant homeostasis and lung cancer progression.

Next, we successfully achieved overexpression of CBS and GCLC in DDX3X-deficient cells (Fig. 3I, J). Notably, the overexpression of CBS nearly restored the growth impairment seen in DDX3X-knockdown cells, while GCLC overexpression had a less pronounced effect on the proliferation of DDX3X-deficient cells (Fig. 3K and Fig. S5I–K). Consistent with this, cysteine supplementation—synthesized by CBS—enhanced the viability of DDX3X-deficient cells but had little impact on DDX3X-competent cells (Fig. 3L). Interestingly, cysteine was more effective at restoring the viability of DDX3X-knockdown cells under stressed conditions (Fig. 3M, N). Moreover, the provision of cysteine-enriched drinking water expedited lung tumor progression solely in *KP;Ddx3x*^*fl/y*^ mice but not in their *KP; Ddx3x*^*+/y*^ littermates (Fig. 3O). Thus, these findings underscore CBS-mediated cysteine metabolism as a pivotal downstream effector in DDX3X-driven lung cancer progression.

### DDX3X controls the translation of CBS through METTL16

**Fig. 4:**
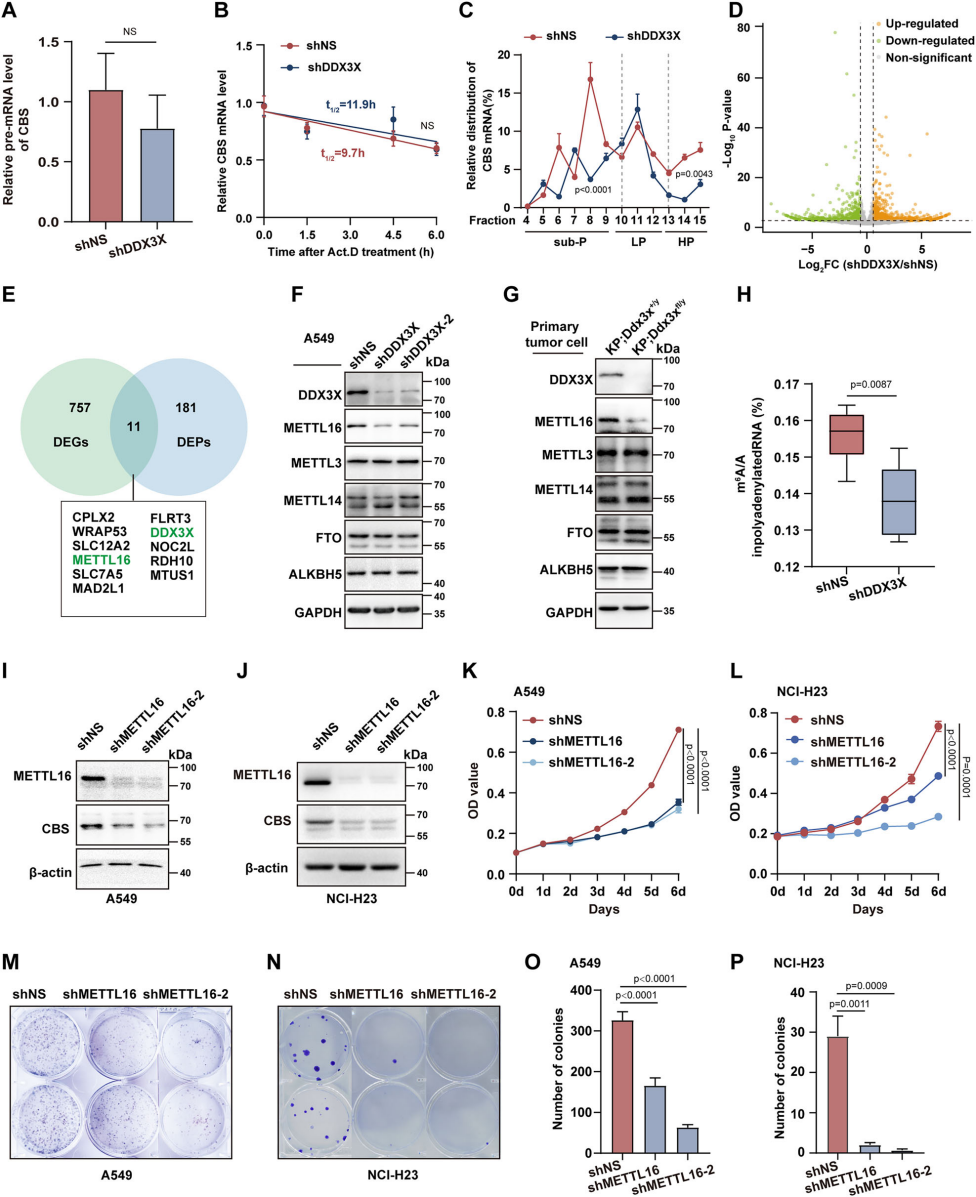
DDX3X controls the translation efficacy of CBS through METTL16. **A** Quantitative real-time PCR analysis of pre-mRNA levels measured by in shNS or shDDX3X A549 cells (*n* = 3, mean ± SEM, NS. *p* > 0.05, unpaired Student’s t test). **B** Assessment of *CBS* mRNA stability in shNS or shDDX3X A549 cells (*n* = 5, mean ± SEM, NS. *p* > 0.05, unpaired Student’s t test). **C** Polysomal analysis of *CBS* mRNA in shNS or shDDX3X A549 cells, mean CBS mRNA abundance in each polysomal fraction was presented (*n* = 3, mean ± SEM, One-way ANOVA). **D** Volcano plot from RNA-seq data showing downregulated mRNAs (fold change <0.6 and *p-*value <0.05, green dots) and upregulated mRNAs (fold change >1.5 and *p*-value <0.05, orange dots) in A549 cells upon DDX3X knockdown. **E** Venn diagram highlights overlaps between downregulated transcripts and proteins in shDDX3X A549 cells compared with shNS control cells. **F**, **G** Representative western blot analysis of DDX3X, METTL16, METTL3, METTL14, FTO, and ALKBH5 in DDX3X knockdown A549 cells **(F)** and DDX3X knockout primary lung cancer cells **(G)**. GAPDH was used as loading control. **H** Analysis of the m^6^A-to-A ratio in mRNAs from shNS or shDDX3X A549 cells, measured by LC-MS/MS (n = 6, mean ± SEM, One-way ANOVA). **I**, **J** Representative analysis of METTL16 and CBS protein level in METTL16-knockdown A549 cells **(I)** and NCI-H23 cells **(J)**. **K**, **L** A 6-day period proliferation assay of control or METTL16 knockdown A549 cells **(K)** and NCI-H23 cells **(L)** (n = 3, mean ± SEM, Two-way ANOVA). **M**, **N** Colony formation assay in control or METTL16 knockdown A549 cells **(M)** and NCI-H23 cells **(N)**, cell colonies were stained and assessed after a 2-week incubation period. **O**, **P** Quantification of colony formation in **(M)** and **(N)**, respectively. (*n* = 3, mean ± SEM, unpaired Student’s t test).

To elucidate how DDX3X governs CBS expression, we initially assessed the production of nascent *CBS* RNA in DDX3X knockdown and control cells. Quantitative real-time polymerase chain reaction (qRT-PCR) with primers spanning intron-exon boundaries revealed that DDX3X had no discernible impact on the generation of nascent *CBS* transcripts (Fig. 4A). Furthermore, DDX3X did not influence the stability of *CBS* mRNA (Fig. 4B). Intriguingly, polysome profiling indicated a significant impairment in the translation of *CBS* mRNA in DDX3X-deficient cells (Fig. 4C). To unravel the mechanism of DDX3X-mediated *CBS* translation, we conducted RNA sequencing in DDX3X knockdown cells, revealing 768 downregulated genes and 1050 upregulated genes (Fig. 4D). Integrating the RNAseq with the proteomics data, focusing specifically on downregulated genes/proteins, we identified 11 genes that exhibited alterations at both the transcriptional and protein levels (Fig. 4E). Among these, METTL16, a methyltransferase involved in RNA m^6^A modification [34, 35] and metabolism [36], emerged as significantly downregulated in the absence of DDX3X (Fig. 4E). Intriguingly, the expression of other crucial m^6^A regulators, including METTL3, METTL4, FTO, and ALKBH5, remained unaffected in DDX3X-deficient A549, NCI-H23 cells and primary lung tumor cells derived from *KP;Ddx3x*^*fl/y*^ mice (Fig. 4F, G, Fig. S6A). Consistent with these findings, liquid chromatography-tandem mass spectrometry (LC-MS/MS) was employed to assess the m^6^A modification level of total mRNA, revealing a marked decrease in global m^6^A modification in DDX3X-knockdown cells (Fig. 4H). To establish a direct link between METTL16 and CBS, we knocked down METTL16 in A549 and NCI-H23 cells, resulting in clear inhibition of CBS expression (Fig. 4I, J) and cell proliferation (Fig. 4K–P). These findings collectively suggest that METTL16 plays a pivotal role in mediating the regulation of CBS expression by DDX3X.

### METTL16-dependent m^6^A modification is required for DDX3X-regulated CBS expression and lung cancer proliferation

**Fig. 5:**
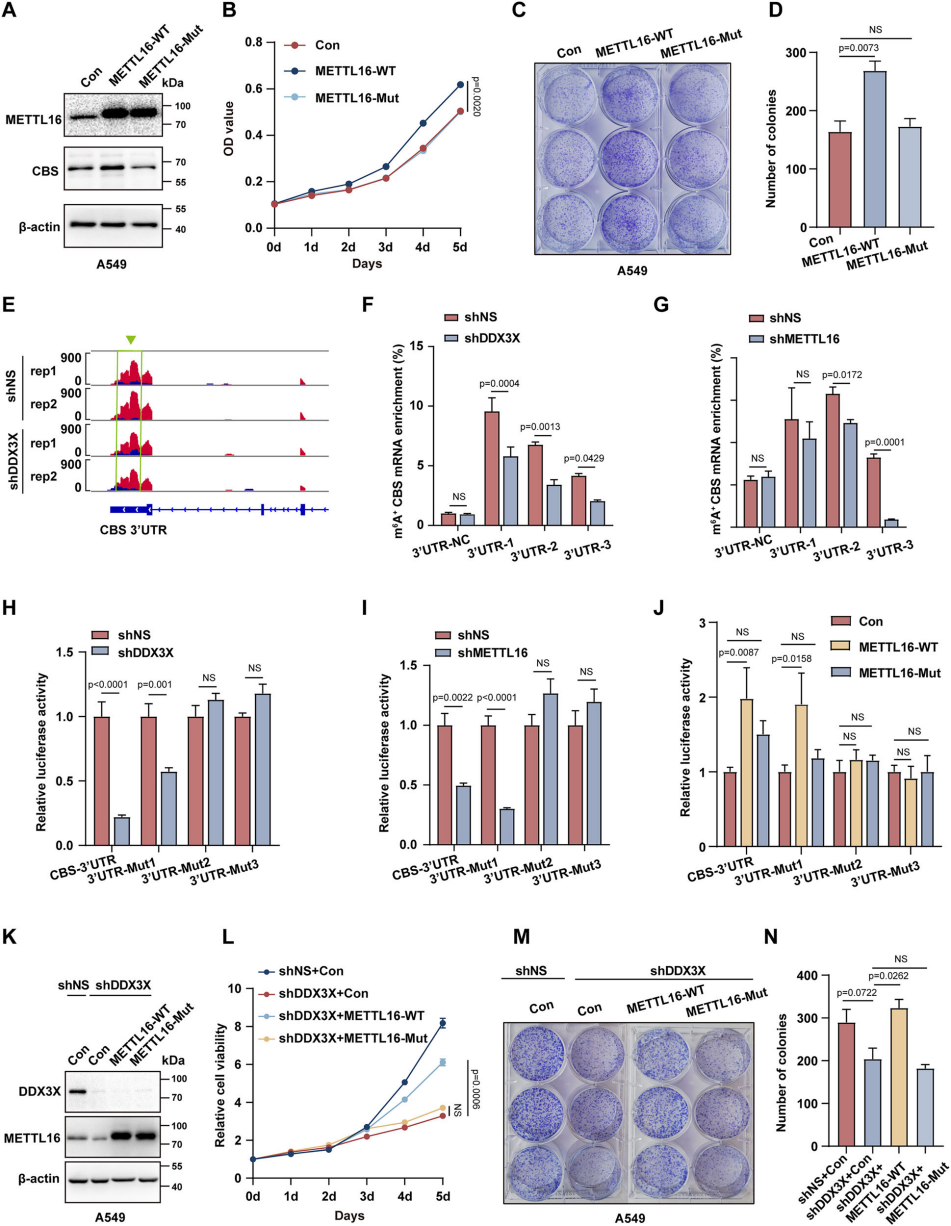
METTL16-dependent m^6^A modification is required for DDX3X regulated CBS expression and lung cancer proliferation. **A** Protein expression of METTL16, and CBS in overexpressed WT or Mut METTL16 A549 cells. β-actin was employed as a loading control. **B** A 5-day period proliferation assay of overexpressed WT or Mut METTL16 A549 cells (*n* = 3, mean ± SEM, NS. p > 0.05, Two-way ANOVA). **C** Representative crystal violet-stained plates depicting the clonogenic capacity of A549 cells with wild-type (WT) or mutant (Mut) METTL16 expression. **D** Quantification of colony formation in **(C). E** IGV snapshots of MeRIP-seq reads along *CBS* mRNAs, showcasing differentially methylated sites in shDDX3X compared to shNS A549 cells. Red peaks indicate methylated sites. Dark peaks indicate input. **F**, **G** m^6^A-RIP-qPCR analysis of CBS 3’ UTR fragments. Quantitative assessment of m^6^A modification in CBS 3’UTR fragments in DDX3X knockdown **(F)** or METTL16 knockdown **(G)** A549 cells (*n* = 3, mean ± SEM, NS. *p* > 0.05, unpaired Student’s t test). **H-J** Evaluation of luciferase reporter activity in DDX3X knockdown **(H)**, METTL16 knockdown **(I)** and overexpressed WT or Mut METTL16 **(J)** A549 cells with different 3’UTR mutants, Renilla luciferase activity served as an internal control (*n* = 3, mean ± SEM, NS. *p* > 0.05, unpaired Student’s t test). **K** Evaluation of METTL16 and DDX3X expression in A549 cells transduced with lentiviral shNS or shDDX3X as well as overexpressed WT or Mut METTL16. β-actin was employed as a loading control. **L** Quantification of relative cell viability in A549 cells transduced with shNS or shDDX3X as well as overexpressed WT or Mut METTL16 (*n* = 3, mean ± SEM, NS. *p* > 0.05, Two-way ANOVA). **M** Colony formation assay of A549 cells transduced with shNS or shDDX3X as well as overexpressed WT or Mut METTL16, cell colonies were stained and evaluated after a 2-week incubation period**. N** Quantification of colony formation in **(M)**.

To investigate the correlation between METTL16 expression and lung cancer patient prognosis, we conducted a comprehensive analysis of survival data from lung cancer cases profiled by TCGA. Notably, elevated METTL16 expression was associated with poorer overall survival and progression-free intervals in lung adenocarcinoma (LUAD) (Fig. S6B, C). Next, we wondered whether METTL16-mediated m^6^A modification was required for DDX3X-regulated CBS expression. Thus, a mutant variant of METTL16 (devoid of m^6^A modification activity) (Table S5) was established in A549 cells. Notably, only overexpression of wildtype METTL16, but not the mutant METTL16 led to elevated CBS expression (Fig. 5A). As expected [37], only overexpressing wildtype METTL16 triggers lung cancer cells proliferation (Fig. 5B–D). With strict peak-calling criteria, we observed altered m^6^A peaks in shDDX3X as compared to shNS A549 cells, with a predominant clustering toward the boundary between the 3’UTR and CDS (Fig. S6D–F). Notably, these modified peaks were enriched for the canonical DRACH motif associated with sites of m^6^A modification (Fig. S6G).

Importantly, hypomethylated differential peaks were observed in the CBS 3’UTR region affected by DDX3X knockdown (Fig. 5E and Fig. S6H). Using the sequence-based m^6^A modification site predictor SRAMP [38], we evaluated the m^6^A level of 3 sites (3’UTR-1, 3’UTR-2, 3’UTR-3) in the 3’UTR domain of CBS mRNA and observed a significant decreased m^6^A modification in all these sites in DDX3X knockdown cells (Fig. 5F), similar phenotype was also found in METTL16 knockdown cells (Fig. 5G). To examine which m^6^A modification sites were required for CBS expression, we generated 3 mutants using dual-luciferase reporter vectors containing every segment of CBS 3’UTR with different m^6^A modification sites’ mutation (Fig. S6I). Our data suggested that mutation of 3’UTR-1 had no obvious effect to rescue the phenotype caused by DDX3X or METTL16 deficiency, whereas mutation of 3’UTR-2 or 3’UTR-3 fully eliminated the outcome induced by DDX3X or METTL16 inhibition (Fig. 5H, I), suggesting these two m^6^A modification sites were essential to mediate the METTL16 regulation on CBS expression. In line with this, overexpression of wildtype METLL16 enhanced the expression of CBS with 3’UTR-1 mutation but not with 3’UTR-2 or 3’UTR-3 mutation, further confirming the importance of these two sites in controlling CBS expression (Fig. 5J). Finally, we introduced overexpression of either wildtype or mutant METTL16 in different DDX3X knockdown cells (Fig. 5K), and only wildtype METTL16 could restore the proliferation and growth disadvantage in DDX3X deficient cells (Fig. 5L–N, Fig. S6J, K).

### DDX3X orchestrates *METTL16* transcription through direct interaction with *JUND* transcripts

**Fig. 6:**
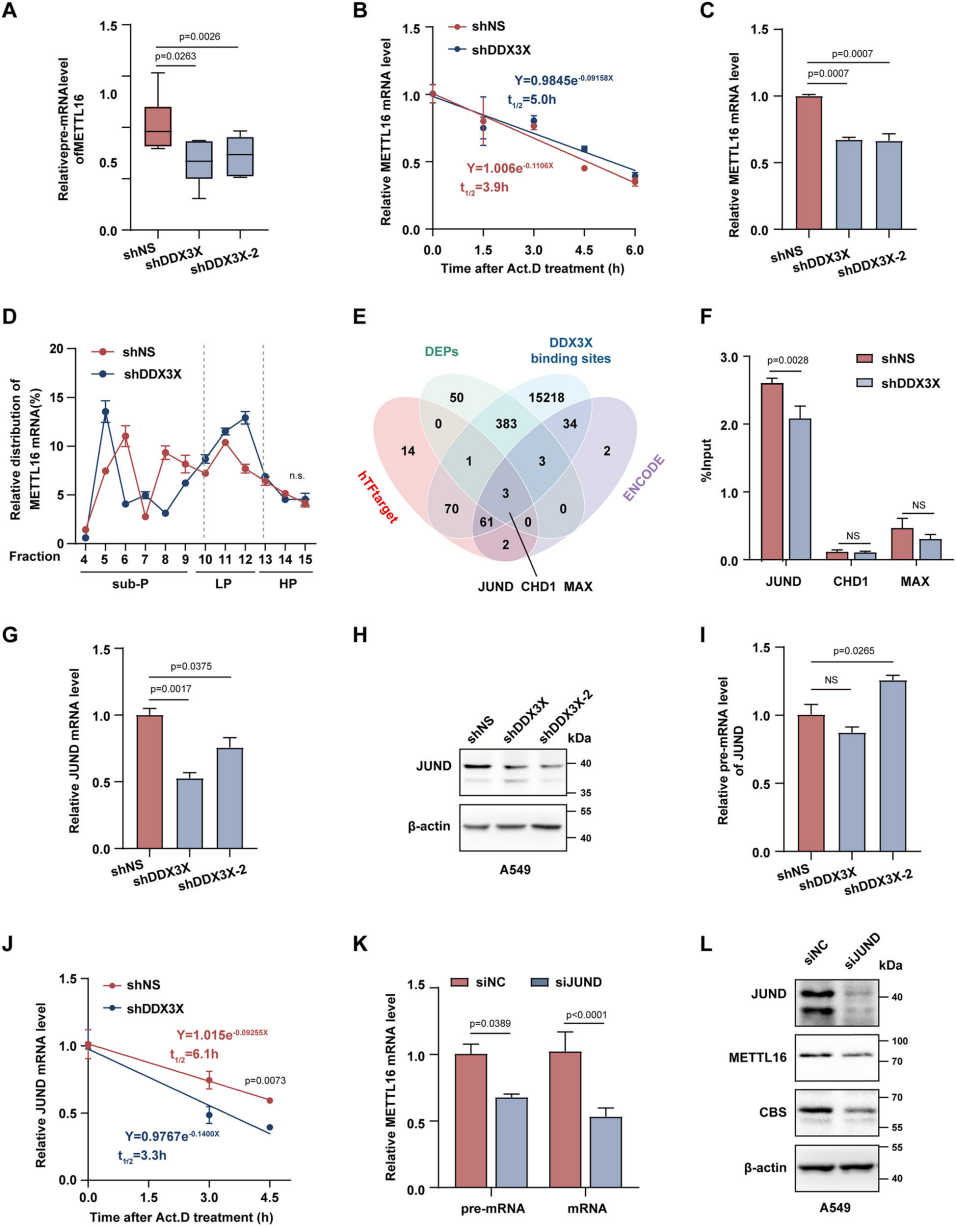
DDX3X regulates *METTL16* transcription through direct interaction with *JUND* transcripts. **A** Quantification of *METTL16* pre-mRNA in A549 cells transduced with shNS or shDDX3X (*n* = 3, mean ± SEM, One-way ANOVA). **B** Degradation assay of *METTL16* mRNA evaluated by qRT-PCR in shNS or shDDX3X A549 cells treated with Actinomycin D (*n* = 3, mean ± SEM, NS. p > 0.05, Two-way ANOVA). **C** qRT-PCR analysis of *METTL16* total mRNA in control or DDX3X knockdown A549 cells (*n* = 3, mean ± SEM, One-way ANOVA). **D** Mean abundance of *METTL16* mRNA in each polysome fraction from polysomal analysis conducted in shNS and shDDX3X A549 cells (*n* = 3, mean ± SEM, NS. p > 0.05, One-way ANOVA). **E** Venn diagram showing the distribution of transcripts among the CHIP dataset of transcription factors for METTL16 (hTFtarget or ENCODE database), DEPs (differential proteins upon DDX3X-knockdown), and DDX3X-directly bound transcripts (RBPDP database). The shared transcripts among all these datasets were highlighted. **F** Enrichment of *JUND*, *CHD1*, and *MAX* mRNA was assessed through an anti-DDX3X immunoprecipitation assay followed by qRT-PCR with normalization to input mRNA levels (*n* = 3, mean ± SEM, NS. *p* > 0.05, unpaired Student’s t test). **G** qRT-PCR assay of relative levels of *JUND* mRNA in shNS or shDDX3X A549 cells (*n* = 3, mean ± SEM, One-way ANOVA). **H** Representative JUND expression in shNS or shDDX3X A549 cells. **I** qRT-PCR assay of relative *JUND* pre-mRNA levels in shNS or shDDX3X A549 cells (*n* = 3, mean ± SEM, NS. *p* > 0.05, One-way ANOVA). **J** Degradation assay of *JUND* mRNA analyzed by qRT-PCR in shNS or shDDX3X A549 cells treated with Actinomycin D (*n* = 3, mean ± SEM, Two-way ANOVA). **K** Relative pre-mRNA and mRNA levels of *METTL16* in control or JUND knockdown A549 cells (*n* = 3, mean ± SEM, unpaired Student’s t test). **L** Representative expression of JUND, METTL16, and CBS in control and JUND knockdown A549 cells.

To elucidate the mechanism by which DDX3X controls METTL16 expression, we initiated a study employing qRT-PCR primers designed to span both introns and exons of *METTL16* [39], facilitating the detection of nascent RNA production (Fig. S7A). Notably, a significant reduction in nascent *METTL16* RNA levels was observed in DDX3X knockdown A549 and PC-9 cells (Fig. 6A and Fig. S7B). Intriguingly, there was no significant difference in the decay of *METTL16* mRNA when DDX3X was deficient (Fig. 6B). Importantly, the overall *METTL16* mRNA levels exhibited a decrease in DDX3X knockdown A549 cells and PC-9 cells (Fig. 6C and Fig. S7C), with no obvious alterations in *METTL16* translation efficiency in the absence of DDX3X (Fig. 6D). These findings strongly suggest that DDX3X regulates METTL16 expression at the transcriptional level. To delve into the regulatory mechanism of DDX3X over *METTL16* transcription, we conducted an integrated study, combining differentially expressed proteins (DEPs) from our proteomics datasets, DDX3X-binding transcripts from the POSTAR3 database, and predicted transcription factors for METTL16 using both hTFtarget and ENCODE databases. Through this approach, two downregulated transcription factors (JUND, CHD1) [40, 41] and one upregulated transcription factor (MAX) [42] were identified as direct binding targets of DDX3X involved in METTL16 transcriptional regulation (Fig. 6E). Next, RNA immunoprecipitation (RIP) followed by qRT-PCR analyses revealed a significant enrichment of the *JUND* transcript with DDX3X protein, confirming the direct interaction between *JUND* mRNA and DDX3X protein endogenously (Fig. 6F). Notably, knocking down of DDX3X resulted in a reduction in JUND expression at both the mRNA and protein levels (Fig. 6G, H), however, DDX3X inhibition had no effect on nascent *JUND* transcript production but significantly impaired the stability of *JUND* mRNA (Fig. 6I,J). Given that JUND is a transcription factor in lung cancer progression [43], we further investigated whether JUND acts as a nexus between METTL16 and DDX3X. Silencing JUND with siRNA in A549 and PC-9 cells clearly reduced METTL16 nascent RNA, mRNA production, and protein expression (Fig. 6K, L, Fig. S7D, E). Finally, by performing co-immunoprecipitation and immunofluorescence assay, we excluded the possibility of direct interaction between DDX3X and JUND in lung cancer cells (Fig. S7F–I). These observations substantiate our hypothesis that JUND plays a crucial role in mediating the transcriptional regulation of METTL16 by DDX3X.

Finally, we evaluated the prognostic value of DDX3X and its associated genes by interrogating DDX3X/CBS, DDX3X/METTL16 and DDX3X/JUND respectively using TCGA. Our data indicated that patients with higher expression of DDX3X/CBS, DDX3X/METTL16 and DDX3X/JUND exhibited poorer overall survival and progression-free interval (Fig. S8A–F), suggesting the prognostic value of DDX3X and its associated genes for lung cancer patients.

### The DDX3X degrader J10 shows enhanced cytotoxic activity relative to RK-33

**Fig. 7:**
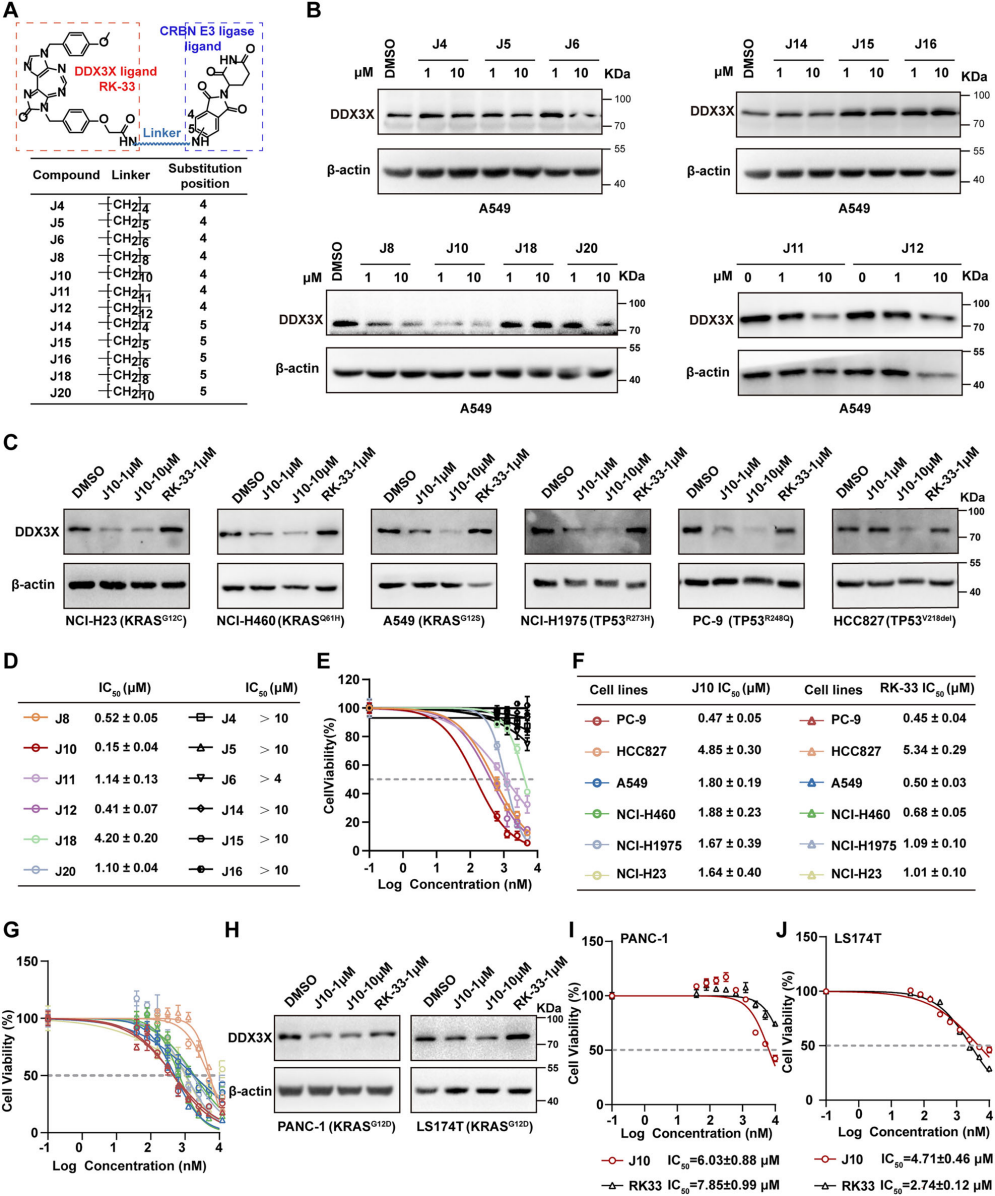
The DDX3X degrader J10 exhibits anti-tumor activity against lung cancer. **A** Chemical structures of PROTAC DDX3X degraders J4, J5, J6, J8, J10, J11, J12, J14, J15, J16, J18, J20. **B** Evaluation of DDX3X protein levels in A549 cells treated with different doses of PROTAC DDX3X degraders for 48 h (*n* = 3). **C** Immunoblot analysis of DDX3X in PC-9, HCC827, A549, NCI-H460, NCI-H1975, and NCI-H23 cells after treatment with J10, RK-33 or DMSO for 24 h. **D** Calculated IC_50_ values from dose response assays in **(E). E** Representative concentration-response curves were determined by CCK8 assay after treatment with different PROTAC DDX3X degraders for 48 h (*n* = 3, mean ± SEM). **F** Display of IC_50_ values of **(G)**. Data represent the mean ± SD from three biological replicates (*n* = 3). **G** IC_50_ analysis of J10 and RK-33 in indicated human lung cancer cell lines for 24 h. **H** Immunoblot analysis of DDX3X protein level after 24 h treatment with J10, RK-33, or DMSO (vehicle control) in PANC-1 and LS174T cells. β-Actin was used as loading control. **I**, **J** Dose-response curves and calculated half-maximal inhibitory concentrations (IC_50_) of J10 and RK-33 in PANC-1 pancreatic ductal adenocarcinoma **(I)** and LS174T colorectal adenocarcinoma cell lines **(J)** (both harboring KRAS^G12D^ mutations) after 24 h treatment.

In our pursuit of evaluating DDX3X as a therapeutic target in lung cancer, we conceived twelve potential heterobifunctional DDX3X degraders using Proteolysis Targeting Chimeras (PROTACs) technology [44], which leverage the ubiquitin-proteasome system to induce DDX3X degradation (Fig. 7A). Drawing inspiration from the reported binding modes of the known DDX3X inhibitor RK-33 [11, 23], which demonstrated that the methoxybenzyl group on the imidazolone moiety positioned toward the solvent-exposed region could serve as a starting point for molecular design. We connected RK-33 to cereblon (CRBN) ligand thalidomide, generating PROTAC DDX3X degraders with linker arms of varying lengths for the investigation of DDX3X protein-degrading activity and antitumor efficacy. The synthesis of the diimidazolodiazepine DDX3X PROTAC degraders used in this study is outlined (Fig. S9A). Our initial assessment of DDX3X protein degradation efficacy revealed that among the compounds tested, J10 exhibited superior degrading activity compared to others, suggesting a preference for a linker with a suitable length at the 4-position of thalidomide (Fig. 7B). In addition, we showed that J10 efficiently degraded DDX3X in all 6 lung cancer cell lines tested in this study (Fig. 7C). Subsequently, we evaluated the impact of these compounds on lung cancer cell viability in A549 cell lines. Notably, J8, J10, J11, J12, J18, and J20 demonstrated the ability to suppress lung cancer cell growth, with IC_50_ values of 0.52, 0.15, 1.14, 0.41, 4.2, and 1.1 μM, respectively (Fig. 7D, E). To ensure these effects were not cell-line specific, we investigated the impact of J8 and J10 in two other lung cancer cell lines with elevated DDX3X expression, NCI-H460 and NCI-H1975, obtaining similar results (Fig. S9B–D). However, the overall cytotoxic effects of RK-33 and J10 were comparable in these lung cancer cell lines (Fig. 7F, G). Given that KRAS^G12D^ is the most frequent KRAS mutation in both pancreatic and colorectal cancers, we further extended our analysis to include KRAS^G12D^-mutant pancreatic (PANC-1) and colorectal (LS174T) cell lines. Consistent with our previous findings, J10 treatment effectively decreased DDX3X protein levels (Fig. 7H) and showed comparable cytotoxicity with RK-33 in pancreatic cancer and colon cancer (Fig. 7I, J).

### The DDX3X degrader J10 exhibits superior tumor-suppressive effects and enhances radiosensitivity compared to RK-33

Our next objective was to compare the anti-tumor efficacy of J10 with RK-33, a small molecule DDX3X inhibitor that has been shown to have anti-tumor effects [23]. We first evaluated the therapeutic potential of J10 using an A549 xenograft model, which demonstrated significant tumor growth inhibition when administered at 10 mg/kg every other day (Fig. S10A–E). Next, we test the antitumor efficacy of J10 in spontaneous *Kras*^*G12D*^*; p53*^*fl/fl*^ lung cancer model, where J10 treatment significantly extended overall survival and markedly delayed disease progression (Fig. S10F, G). Additionally, J10 exhibited robust antitumor effects in lung cancer patient-derived organoids (PDOs) (Fig. S10H, I).

**Fig. 8:**
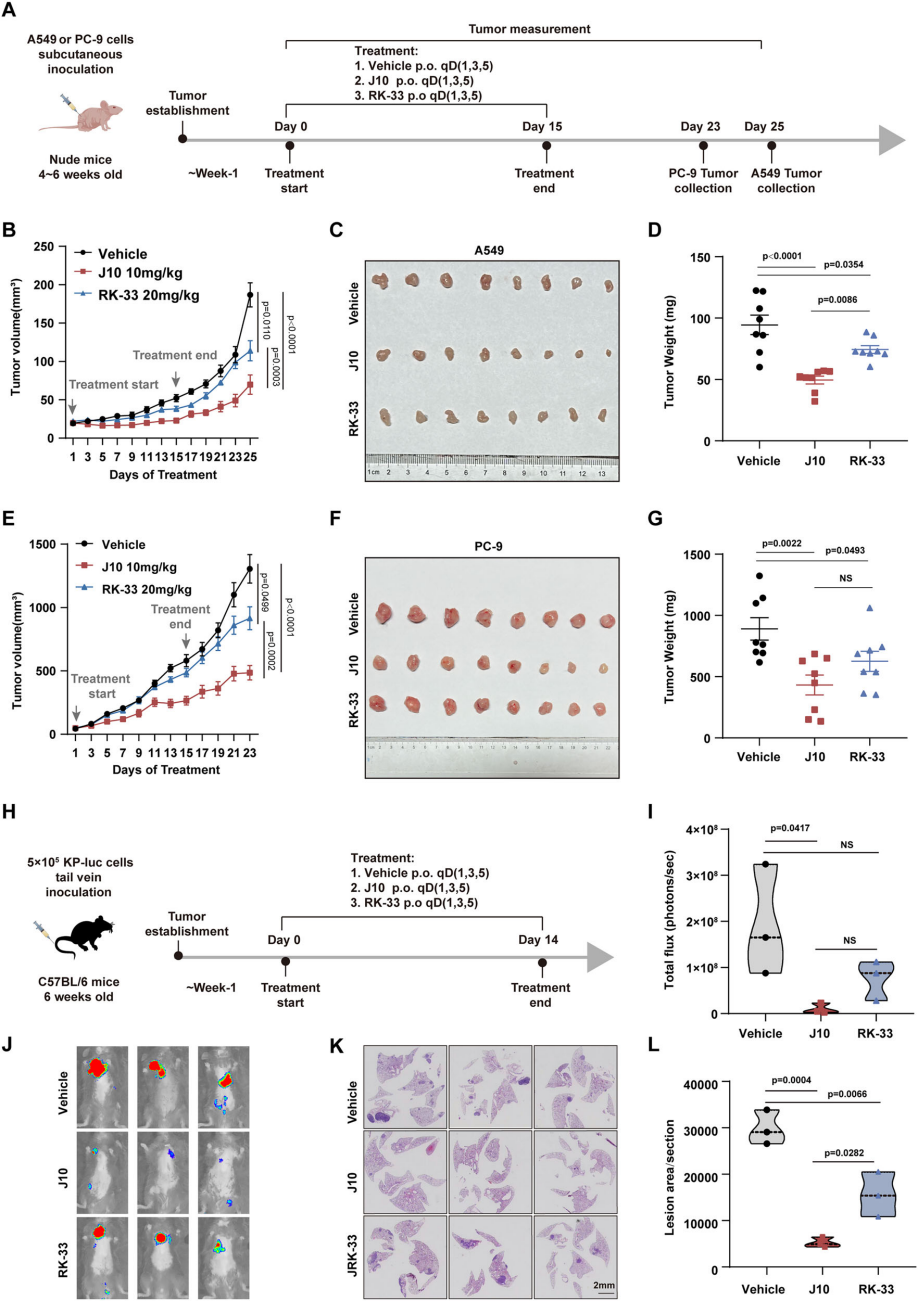
The DDX3X degrader J10 shows superior tumor suppressing effect compared to RK-33 in vivo. **A** Schema for the pharmacodynamic (PD) study in A549 and PC-9 xenografts. BALB/c nude mice bearing subcutaneous xenografts of A549 (5 × 10^6^ cells) or PC-9 (2.5 × 10^6^ cells) were treated with 10 mg/kg J10, 20 mg/kg RK-33 or vehicle via oral gavage (p.o.) on day 1, 3, and 5 each week for 2 weeks and day 1 for the third week. Mice were continuously monitored for 10 days after the last treatment. *n* = 8 mice per group were used. **B-D** The tumor growth curves of A549 xenograft tumors (**B),** representative images of A549 xenograft tumors (**C)** and tumor weight analysis of A549 xenograft tumors (**D)** treated with 10 mg/kg J10, 20 mg/kg RK-33 or vehicle. Data was presented as mean ± SEM. The tumor growth curves were assessed using two-way ANOVA, tumor weight was assessed using one-way ANOVA, *n* = 8 mice per group. **E-G** The tumor growth curves of PC-9 xenograft tumors **(E)**, representative images of PC-9 xenograft tumors **(F)** and tumor weight analysis of PC-9 xenograft tumors **(G)** treated with 10 mg/kg J10, 20 mg/kg RK-33 or vehicle. Data was presented as mean ± SEM. The tumor growth curves were assessed using two-way ANOVA, tumor weight was assessed using one-way ANOVA, n = 8 mice per group. **H** Schema for the in vivo tumor suppressing analysis using KP tumor cells (*KRAS*^*G12D*^*;p53*^*-/-*^ derived from mouse lung adenocarcinoma) in C57BL/6 mice. 5×10^5^ KP-luc (luciferase) cells was intravenously (through the tail vein) injected into C57BL/6 mice. One week after injection, mice were treated with J10 (10 mg/kg), RK-33 (20 mg/kg), or vehicle via oral gavage (p.o.) three times per week for two weeks. After 14 days of treatment, three mice from each group (vehicle, J10, and RK-33) were harvested for analysis. **I** Quantification of flux (photons/sec) with an exposure time of 60 s in **(J)**. *n* = 3, mean ± SEM with ordinary One-way ANOVA. **J** Representative bioluminescence images of mice obtained 14 days after treatment with vehicle, J10, or RK-33. **K** Representative images of H&E-stained lung tissue sections from mice treated with vehicle, J10, or RK-33 for 14 days. Scale bar: 2 mm. **L** Quantification of the lesion area in sections shown in **(K)**. *n* = 3, mean ± SEM with ordinary One-way ANOVA.

We then compared the therapeutic potential of J10 and RK-33 in different mouse lung tumor models. In subcutaneous models with A549 and PC-9 cells, J10, administered orally at 10 mg/kg, significantly outperformed RK-33 at 20 mg/kg in suppressing tumor proliferation, growth, and weight (Fig. 8A–G). Additionally, in an orthotopic lung cancer model, established by injecting *KRAS*^*G12D*^*;p53*^*-/-*^ luciferase-expressing cells into the tail vein, J10 also exhibited a superior tumor suppression effect compared to RK-33 under the same treatment regimen (Fig. 8H–L). We also assessed the potential toxicity of RK-33 and J10 in human normal lung epithelial cells BEAS-2B. Our results indicated that J10 had only a mild degradative effect in BEAS-2B cells (Fig. S11A), and J10 showed significantly lower cytotoxicity compared to RK-33 (Fig. S11B), suggesting that J10 has a superior safety profile. Remarkably, J10 did not induce significant weight loss (Fig. S11C) or hepatotoxicity (Fig. S11D). Administering J10 did not cause any systemic toxicity, as determined through hematology analyses. Specifically, there were no significant changes observed in the counts of platelets, red or white blood cells, lymphocytes, monocytes, neutrophils, or eosinophils in mice treated with J10 compared to control mice (Fig. S11E–L). Intriguingly, J10 showed similar capacity to induce radiosensitization as RK-33 in lung cancer cells (Fig. S12A–D). These data demonstrate that J10 exhibits superior and more sustained tumor-suppressive effects compared to RK-33 in vivo.

## Discussion

Despite the growing recognition of the critical role of RNA metabolism in various pathological processes [45], many members of the DDX (DEAD-box RNA helicase) family lack established gene-disease associations, particularly in cancer progression [46, 47]. However, DDX3X has garnered significant attention in recent years and is emerging as a potential therapeutic target for cancer therapy due to its frequent mutations and altered expression in a range of malignancies [48, 49], including prostate cancer [19], pancreatic cancer [50], colorectal cancer [13], head and neck squamous cell carcinoma (HNSCC) [51], oral squamous cell carcinoma [52], breast cancer [53], hepatocellular carcinoma [54], lymphomagenesis [20], and lung cancer [22, 23]. While previous studies have highlighted DDX3X multifaceted roles in lung cancer progression and its potential as a therapeutic target, a comprehensive understanding of its involvement in lung cancer necessitates further in-depth investigations.

Given that *KRAS* mutations are prevalent in almost 30% of non-small cell lung cancer (NSCLC) patients [2], we chose a *Kras*^*G12D*^-driven spontaneous lung cancer murine model to elucidate the detailed mechanism by which DDX3X regulates lung cancer progression, which might be also extended to *KRAS* wildtype lung cancers. Specifically, our study focuses on how DDX3X regulates lung cancer metabolism. Our findings reveal that DDX3X serves as a key regulator of cysteine and glutathione metabolism, thereby controlling ferroptosis in lung cancer cells. This discovery establishes a direct link between DDX3X, metabolism, and cell death in lung cancer.

In our subsequent investigations, we sought to elucidate how DDX3X regulates CBS expression. Given DDX3X role as a crucial RNA-binding protein governing various aspects of gene expression [8, 10, 55], our study in KRAS-driven lung cancer cells revealed that DDX3X influences CBS translation without affecting transcription or mRNA stability. Upon examining genes downregulated by DDX3X inhibition, our integrated analysis pinpointed a methyltransferase, METTL16, known for its involvement in RNA m^6^A modification [34–36]. Subsequent analyses confirmed a global downregulation of m^6^A in DDX3X knockdown cells. Notably, among key m^6^A regulators, only METTL16 expression was suppressed in DDX3X knockdown cells. Furthermore, we demonstrated that METTL16-dependent m^6^A modification is essential for DDX3X-regulated CBS expression and lung cancer proliferation. This represents one of the first pieces of evidence [52] showing that DDX3X can directly regulate m^6^A modification via METTL16. Lastly, we uncovered that DDX3X controls *METTL16* transcription through a direct interaction with *JUND* transcripts, thus delineating an intact pathway elucidating how DDX3X regulates lung cancer progression. These findings add a new perspective on metabolic control to supplement the multifaceted roles of DDX3X in regulating cancer progression. However, we acknowledge the possibility of additional functions of DDX3X in tumorigenesis, which requires further investigations.

While previous studies have highlighted the significance of DDX3X in lung cancer progression [22], and small molecule inhibitors such as RK-33 have been developed for treating lung cancer [23], no clinical trials involving DDX3X inhibitors have been initiated thus far. One potential reason for this could be the concern that inhibiting DDX3X RNA helicase activity might lead to severe side effects. In this study, we have developed a novel DDX3X degrader, J10, using the PROTAC strategy. PROTAC degraders offer several advantages over traditional small molecule inhibitors, including its intrinsic event-driven pharmacology and nonlinear pharmacodynamics, improved safety profiles, and reduced risk of drug resistance. Indeed, using different lung cancer models, we demonstrated that the DDX3X degrader J10 efficiently induces DDX3X degradation and exhibits significantly more anti-tumor effects in all in vivo models with less toxicity compared to RK-33. Therefore, we propose that inducing DDX3X degradation may be a safer approach than inhibiting DDX3X activity, as it might allow for DDX3X to perform its essential functions within a controlled time window. This strategy holds greater potential for future clinical translation.

Our study has several limitations. DDX3X plays a crucial role in maintaining cell survival and growth, thus generating a DDX3X knockout cell line is challenging, as complete elimination of endogenous DDX3X is unattainable. Furthermore, upon inhibition of DDX3X, we observed an upregulation of DDX3Y expression, a homologous gene located on the Y chromosome (Fig. S12E–H). This phenomenon could potentially compromise the efficacy of DDX3X inhibition. However, due to the lack of a specific antibody for DDX3Y, we were unable to examine the distinctions between DDX3X and DDX3Y in this study. While our investigation highlights one mechanism by which DDX3X regulates lung cancer progression, it does not preclude the existence of other potential roles of DDX3X in tumorigenesis. Finally, the applicability of our findings to other tumor types necessitates further investigation.

## Materials and methods

### Cell lines

The A549, PC-9, NCI-H460, NCI-H1975, HCC827, NCI-H23, PANC-1, LS174T, HEK293T, and BEAS-2B cells, were purchased from the China Center for Type Culture Collection (CCTCC). A549 and BEAS-2B cells were cultured in Ham’s F-12K Medium, while PC-9, NCI-H460, NCI-H1975, HCC827and NCI-H23 tumor cells were maintained in RPMI 1640 Medium. HEK293T, PANC-1, LS174T cells were cultured in high glucose Dulbecco’s modified Eagle’s medium (DMEM). The medium was supplemented with 10% heat-inactivated fetal bovine serum (FBS) and 1% Penicillin-Streptomycin (P/S). All cells were confirmed to be free of mycoplasma contamination. Additionally, all cell cultures were maintained at 37°C in a humidified incubator under a 5% CO_2_ atmosphere. All reagent information is detailed in Table S1.

### Animal studies

C57BL/6 and BALB/c nude mice aged 4–6 weeks were purchased from Guangzhou Southern Medical University Laboratory Animal Sci.&Tech.Co. The *Kras*^*LSL-G12D*^*;p53*^*flox/flox*^, and *Ddx3x*^*flox/flox*^ transgenic mice were gifts from the Institute of Molecular Biotechnology of the Austrian Academy of Science (IMBA). Mice were maintained under specific-pathogen free (SPF) conditions in individually ventilated cages at 21–22 °C and 39–50% humidity, with a 12-hour light–dark cycle. All mouse experiments were performed in compliance with the guidelines of the Institutional Animal Care and Use Committee of Southern Medical University Nanfang Hospital and were approved under protocol number IACUC-LAC-20220926-001.

### Subcutaneous implantation of lung cancer xenografts

A total of 5 × 10^6^ A549 or 2.5 × 10^6^ PC-9 cells were subcutaneously injected into the right flanks of 6-week-old male nude mice. One week post-injection, the mice were randomly divided into three groups, with 8 mice per group (24 mice in total): vehicle (group 1), 10 mg/kg J10 (group 2), and 20 mg/kg RK-33 (group 3, Ambeed, A179326). J10 and RK-33 was administered via oral gavage on days 1, 3, and 5 per week for two weeks, followed by a one-week observation period without treatment. Tumor volume was measured regularly using vernier calipers and calculated using the formula [V = ½ (Length × Width²)]. On day 25, mice were anesthetized with 1% pentobarbital, and peripheral blood, tumor tissues, and organs were collected for subsequent analysis.

### Orthotopic model of lung cancer

Stably transfected KP tumor cells expressing luciferase (KP-Luc) were injected into the tail vein of 6-week-old C57BL/6 mice, with each mouse receiving a 100 μL PBS suspension containing 5 × 10^5^ proliferating cells. Seven days post-injection, the mice were randomly assigned to one of three groups: a control group and two treatment groups. The control group received vehicle treatment, while the treatment groups were administered either 10 mg/kg J10 or 20 mg/kg RK-33 via oral gavage on days 1, 3, and 5 per week, continuing until day 21. Body weight was monitored regularly throughout the study. On day 21, tumor progression was evaluated using bioluminescence imaging with the IVIS system (Caliper Life Sciences) following intraperitoneal injection of D-Luciferin potassium salt (3 mg/kg per mouse, administered 5 minutes prior to imaging, Beyotime, ST198) and anesthesia with 1% pentobarbital sodium (80 mg/kg per mouse). At the conclusion of the experiment, peripheral blood and tissues from the lungs, heart, liver, spleen, and kidneys were harvested. Tumor and organ samples were fixed in formalin for histopathological analysis, while a portion of the tumor tissue was cryopreserved for molecular studies. Cellular immunoassays were performed on peripheral blood, and serum samples were analyzed for hepatotoxicity.

### Intranasal adenovirus administration

The Cre-expressing adenovirus (Ad5-mSPC-Cre) utilized in this study was obtained from Genechem Biotech Co., China. Virus administration was carried out via intranasal delivery using previously reported protocols [56]. Briefly, 6- to 8-week-old mice were anesthetized with 1.5% sodium pentobarbital and positioned on a heating pad. The 62.5 μL adenovirus mixture (480 μL Minimum essential medium (MEM), 40 μL adenovirus (3 × 10^10^ TU/ml), and 5.2 μL of 1 M CaCl₂), was then administered intranasally. All viral injections were performed in a biosafety level 2+ facility, strictly following the guidelines set by the Biosafety Committee of Southern Medical University to ensure safety and compliance with biosafety standards. The primers used for genotyping the mice are listed in Table S4.

### Isolation and culture of primary murine lung tumor cells

Primary murine lung cancer cells were isolated and purified using established protocols. Mice, 10-12 weeks after adenovirus infection, were anesthetized, and their thoracic cavities were exposed. Two milliliters of digestive solution [4 mL of 2 μg/mL Dispase (Corning, 354235) and 40 μL of 0.05 mg/mL DNase I (Sigma, DN25)] was then quickly instilled through the trachea into the lungs, after which the trachea was tied off. The lungs (excluding the heart) were carefully removed and incubated in 5 mL of digestive solution in a 15 mL tube for 60–90 min in a 37 °C water bath. The tube was gently agitated every 5–8 min during the incubation to facilitate digestion. Following digestion, the lung tissue was minced using a scalpel blade, and the resulting tissue/cell suspension was filtered twice through a 70 μm strainer while grinding. The filtered suspension was then centrifuged for 4 min at 1000 rcf. After discarding the supernatant, the cell pellet was resuspended in 4 mL of erythrocyte lysis buffer and allowed to stand for 3 min to lyse erythrocytes. After this, 15 mL of DMEM was added, and the cell suspension was filtered again through a 70 μm strainer. The suspension was centrifuged once more, and the cell pellet was resuspended in 10 mL of DMEM. To eliminate immune cells, the cell suspension was incubated in dishes coated with 50 μL of anti-mouse CD16/32 Antibody (Biolegend, 101302, 0.5 mg/mL) for one hour at 37 °C. Non-adherent cells were collected, resuspended in DMEM supplemented with 10% FBS, and transferred to 10 cm culture dishes. These dishes were incubated for one hour at 37 °C to further deplete fibroblasts. Finally, the non-adherent cells were centrifuged again, and the cell pellet was resuspended in 10 mL of culture medium (Ham’s F-12 medium containing 1x GlutaMAX, 15 mM HEPES, 0.8 mM CaCl₂, 0.25% BSA, Insulin-transferrin-selenium (ITS, Sigma, I3146), 2% FBS, and 1% P/S). The cells were then plated onto 10-cm dishes coated with collagen I (Corning, 354236, 200 μg per dish) for further culture.

### Patient lung cancer organoids generation and culture

Patient-derived fresh tissue samples were collected with written informed consent, under the approval of the Institutional Review Board at Nanfang Hospital of Southern Medical University. Organoid lines were developed following established protocols with slight modifications. Fresh tissue samples were washed three times with PBS, placed in sterile 10 cm petri dishes, and mechanically dissected into small pieces before enzymatic digestion. Digestion was performed using 2–3 mL of collagenase media [prepared with 4 mL Dispase (Corning, 354235), 40 μL of 0.05 mg/mL DNase I (Sigma, DN25), 4 μL anti-mouse CD16/32 antibody (Biolegend, 101302), 200 μL collagenase I (Gibco, 17100017), and 10 μM Y-27632 (APExBIO, A3008)]. The mixture was incubated at 37 °C on a shaker at 200 rpm until the solution turned cloudy (approximately 10 minutes). The resulting tissue/cell suspension was filtered through a 70 μm strainer while grinding. To eliminate immune cells, the filtered suspension was incubated in dishes coated with 50 μL of anti-mouse CD16/32 antibody (Biolegend, 101302) for 1 hour at 37 °C. The suspension was centrifuged at 1000 rcf for 3 min, and the cell pellet was resuspended in tissue-specific culture media, including Advanced DMEM/F-12 (Gibco, 12634028) with glutamax (Invitrogen, 35050061), B27 (Gibco, 17504044), 1.25 mM N-acetylcysteine (Macklin, N800425), 50 ng/mL Recombinant Human EGF (R&D, 236-EG-01M), 100 ng/mL Recombinant Human FGF-10 (Peprotech, 100-26-5), 100 ng/mL Recombinant Human FGF-4 (Peprotech, 100-31-25), 100 ng/mL Recombinant Human Noggin (R&D, 6057-NG), 10 μM SB202190 (R&D, 1264), 250 nM CHIR99021 (Sigma, SML1046), anti-mouse CD16/32, 10 μM Y-27632 (APExBIO, A3008), and 500 nM A 83-01(MCE, HY-10432). Up to 50 μL drops of the Matrigel/cell suspension were distributed into a 24-well cell suspension culture plate (Gibco) and allowed to polymerize for 20 min at 37 °C. Tumor-specific primary culture media was then added to each well, with media changed every 3 days. All human lung cancer patient’ samples were proceeded with approval of Medical Ethics committee of Nanfang hospital of Southern Medical University with approve number NFEC-202108-K7.

### Culture of murine lung cancer organoids

5 × 10³ lung tumor cells were mixed with 5 μL of Matrigel (Corning, 354234) and kept on ice until dispensed as rounded drops onto 12-well plates. The plate was incubated at 37 °C for 5–20 min to allow the Matrigel to solidify. Once solidified, 1 mL of primary culture medium was added, and incubation was continued. Images were collected and analyzed after 6 days of incubation.

### Hematoxylin and eosin (H&E) and immunohistochemistry (IHC) staining

H&E staining and IHC staining were performed as previously described. In brief, mouse organs or tumor tissues were fixed with formalin or 4% paraformaldehyde (PFA) respectively overnight, and dehydrated in ethanol, embedded in paraffin, sectioned (4 μm) followed by staining with hematoxylin and eosin. For IHC staining, slides were de-paraffinized in xylene and ethanol, and rehydrated in water. Antigen retrieval was performed by high-pressure heating of slides in sodium citrate buffer (pH 6.0) or 1 mM EDTA buffer for 5 minutes. Slides were quenched in hydrogen peroxide (3%) to block endogenous peroxidase activity. Slides were incubated overnight at 4 °C with primary antibody, rinsed with PBS, and subsequently incubated with HRP-conjugated secondary antibody at 37 °C for 30 min. Following this, slides were stained using a diaminobenzidine (DAB) kit (ZSGB, ZLI-9018) according to the manufacturer’s instructions. Following this, the lung sections were scanned using a Mirax slide scanner. Visual analysis was conducted in a double-blinded manner to ensure unbiased assessment.

### Synthetic PROTAC DDX3X degraders

In the synthesis process, Compound A undergoes a nucleophilic reaction with tert-butyl bromoacetate in the presence of cesium carbonate to yield Compound B. Compounds C and D undergo a nucleophilic reaction in the presence of organic bases to produce Compound E. Subsequently, Compounds B and D undergo tert-butyl and tert-butyloxycarbonyl removal, respectively, in the presence of trifluoroacetic acid. The resulting compounds, F and G, are then evaporated to dryness under reduced pressure. Without purification, Compounds F and G directly undergo a condensation reaction in the presence of a condensing agent and organic base to obtain the diimidazolodiazepine compound described in formula (refer to Fig. 7A) or a pharmaceutically acceptable salt thereof (Fig. S9A).

### Lung tumor histology

Histological analysis was carried out on all lung tumors in accordance with established protocols [57]. In summary, the lungs were sectioned into 4 μm thick slices from at least three different planes and stained with hematoxylin and eosin. Following this, the lung sections were scanned using a Mirax slide scanner, and the lung/tumor areas were automatically scored using an algorithm programmed and executed with the Definiens software suite. Visual analysis was conducted in a double-blinded manner to ensure unbiased assessment.

### siRNAs and shRNA plasmids

To generate constitutive knockdown cell lines, plasmid DNA was purified from pLKO.1 backbone vectors expressing shRNAs targeting DDX3X, CBS, GCLC, METTL16 and a scrambled control. SiRNA was synthesized specifically for transient transfection experiments. The target sequences for shRNA and siRNA are detailed in Table S2.

### Lentiviral production and transduction

Lentivirus production adhered to established procedures. Briefly, HEK293T cells, cultured to approximately 90% confluence on the day of transfection, were subjected to transfection using Lipofectamine 3000. This involved the introduction of packaging plasmids (psPAX2 and pMD2.G) and target plasmids. For every 2 μg of the target plasmid, 18 μL of Lipofectamine 3000 was employed. The cells were incubated with the plasmid/liposome transfection mixture for 8–12 h. Subsequently, the medium was replaced with OPTI-MEM supplemented with 5% FBS and 1% sodium pyruvate. Lentivirus was harvested 72 h post-transfection. For lentiviral transduction, cells were exposed to lentiviral particles for 8–12 h in the presence of polybrene (10 μg/ml) (Santa Cruz Biotechnology).

### In vitro hypoxia and nutrient deprivation model

Cells were cultured to approximately 80% confluence, then the medium was replaced with EBSS containing 200 μM cobalt chloride for the indicated times [55]. For the hypoxia-only model, cells were similarly cultured to 80% confluence and replaced with complete medium containing 200 μM cobalt chloride for the indicated times.

### Cell proliferation assay

Cells were seeded in 96-well plates at a density of 3000–4000 cells per well and allowed to adhere overnight. After incubation, cells were treated with various concentrations of DDX3X degraders for 48 h to assess their inhibitory effects. To compare the efficacy of J10 and RK-33, cells were exposed to different concentrations of each compound for 24 h. The medium was then replaced with 10 μL of CCK-8 solution and 100 μL of culture medium per well. The plates were agitated for 2 h at 37 °C before measuring luminescence. The IC_50_ values were determined using GraphPad Prism.

For the proliferation assay, cells were seeded in 96-well plates at a density of 1000 cells per well and allowed to adhere overnight. Following incubation, 10 μL of CCK-8 solution and 100 μL of culture medium were added to each well. The plates were incubated for 4 h at 37 °C before absorbance was measured at 450 nm to obtain the Day 0 reading. Subsequent readings were taken on different days to monitor cell viability, with each measurement referenced to the initial Day 0 value. All experiments were conducted independently in triplicate.

### RIP-qRT-PCR

RNA immunoprecipitation (RIP) was performed using the Magna RIP Kit (Millipore) following the manufacturer’s instructions. Initially, cells were cultured in 15 cm dishes, washed with ice-cold PBS, and then collected. Subsequently, they were lysed in RIP lysis buffer with added protease and RNase inhibitors. DDX3X antibodies and control mouse IgG (Millipore) were coupled to protein A/G magnetic beads through a 30 min incubation at room temperature. This was followed by three washes and subsequent incubation with cell lysate at 4 °C overnight. After six additional washes, the beads were resuspended, and the proteins were digested using proteinase K at 55 °C for 30 min. The RNA, both input and immunoprecipitated, was extracted and subsequently subjected to either next-generation sequencing (NGS) or qRT-PCR.

### Polysome profiling

Linear sucrose gradients ranging from 5% to 50% were freshly prepared in an ultracentrifuge tube from Beckman, using an automated gradient maker (BioComp). Prior to this, cells were treated with 100 μg/ml Cycloheximide (CHX) (MCE) at 37 °C for 10 min, washed twice with ice-cold PBS containing 100 μg/ml CHX, and then collected. Subsequently, the cells were lysed in a hypertonic buffer comprising 20 mM Tris-HCl (pH 7.4), 300 mM NaCl, 10 mM MgCl_2_, 0.5% sodium deoxycholate, 1% Triton X-100, 1 mM DTT, 100 µg/ml CHX, and 300 U/ml of RNase inhibitor. After lysis, cell debris was removed by centrifugation at 16,000 × g for 10 min at 4 °C. Subsequently, 500 µL of supernatant was loaded into the sucrose gradients, followed by centrifugation at 4 °C and 36,000 rpm for 2 h using the SW41Ti rotor from Beckman. Samples were then fractionated and analyzed using a Piston Gradient Fractionator from BioComp and a fraction collector from Gilson. Briefly, a density gradient fractionation system was used to generate a polysome profile, distinguishing fractions that correspond to 40S (fractions 4 to 6), 60S (fractions 6 to 8), 80S monosomes (fractions 8 to 10), low-molecular-weight polysomes (fractions 10 to 13, LP), and high-molecular-weight polysomes (fractions 13 to 15, HP). For normalization purposes, 5 ng of polyadenylated synthetic luciferase mRNA (Promega) was added to each fraction. RNA was extracted from each fraction and subjected to qRT-PCR analysis.

### Label-free proteome

Begin by resuspending cell pellets in SDT buffer, consisting of 4% SDS and 100 mM Tris-HCl at pH 7.6. The subsequent steps involve protein digestion using trypsin through the Filter-Aided Sample Preparation (FASP) procedure. To each sample, add 40 mM DTT and shake at 37 °C for 1.5 h. Introduce 20 mM IAA, incubate in darkness for 30 min, and mix each sample with 200 μL of 8 M urea before transferring to filters. Centrifuge the filters, wash with 8 M urea buffer three times, followed by two washes with 25 mM NH_4_HCO_3_ buffer. Proceed with trypsin digestion by mixing units with trypsin at a 1:100 ratio. Incubate at 37 °C for 12–18 h, and after the incubation period, spin down the condensate from the filters at 1000 x g for 10 s to collect the resulting peptides. Perform peptide desalting using C18 Cartridges, concentrate peptides through vacuum centrifugation, and reconstitute in 40 µl of 0.1% formic acid. Estimate peptide content by measuring UV light spectral density at 280 nm.

For LC-MS/MS analysis, use the timsTOF Pro mass spectrometry system coupled with Nanoelute. Load peptides onto a C18-reversed phase analytical column with a linear gradient of 99.9% acetonitrile and 0.1% formic acid. Operate the mass spectrometer in positive ion mode with an applied electrospray voltage of 1.5 kV. Employ Parallel Accumulation Serial Fragmentation (PASEF) mode for data collection, with specific parameters, such as ion mobility coefficient (1/K0) set between 0.6 to 1.6 Vs cm2, 1 MS scan, and 10 MS/MS PASEF scans. Combine MS raw data for each sample and analyze using MaxQuant (v1.6.14) software. Utilize specific parameters, including Max Missed Cleavages (2), Fixed modification (Carbamidomethyl-C), Variable modification (Oxidation-M), MS/MS Tolerance (20 ppm), Protein FDR ( ≤ 0.01), Peptide FDR ( ≤ 0.01). Select razor and unique peptides for protein quantification, set a time window (match between runs) of 2 minutes, use LFQ for protein quantification, and ensure a Minimum ratio count of 1.

### MeRIP- qRT-PCR

For the fragmentation of 10 μg of sample RNA, 1 μL of the 10X Fragmentation Buffer (Invitrogen, AM8740) was added to the 10 μL RNA sample. The mixture was thoroughly combined, briefly spun, and incubated at 70 °C for 3 min in a heating block. Subsequently, 1 μL of the Stop Solution was added, and the mixture was placed on ice until further use. Five percent of fragmented RNA was preserved as input, while the remaining RNA underwent m6A-immunoprecipitation (m^6^A-IP) using the EpiMark N6-Methyladenosine Enrichment Kit.For m^6^A-IP, 1 μg of m6A-specific antibody or 1 μg of IgG (Pierce) was added to 40 μL of Protein G Magnetic Beads and incubated with orbital rotation for 1 h with gentle rotation at RT. The fragmented RNA was then divided into two conditions: m^6^A-RIP, incubated with 1 μg of m^6^A-specific antibody, and IgG control, incubated with 1 μg of IgG, rotating head over tail for 20 h with gentle rotation at 4 °C. The bead mixture was eluted with 75 μL of Monarch RNA Cleanup Binding Buffer (0.5 mg/ml N6-Methyladenosine) with gentle rotation at RT for 1 h. Eluted RNA was purified using the Monarch RNA Cleanup Kit. Subsequently, input and IP RNA were utilized for qRT-PCR, as described above. The m^6^A enrichment is determined by calculating the IP/input ratio for the gene of interest, normalizing for expression levels [58]. Specific primers for CBS 3’UTR can be found in Table S3.

### Western blot

Western blotting was conducted following a previously established protocol [59]. Total proteins were extracted from cultured cells using a Total Protein Extraction Kit according to the manufacturer’s instructions. Protein concentrations were determined using a BCA Protein Assay Kit. Lysates were then mixed with the appropriate volume of 5× loading buffer, boiled, and loaded into SDS-PAGE gels for separation. Subsequently, proteins were transferred to polyvinylidene difluoride (PVDF) membranes using a Mini Trans-Blot™ system (Bio-Rad). The membranes were blocked with 5% skim milk in TBST, followed by overnight incubation with primary antibodies at 4 °C, and subsequent incubation with HRP-conjugated secondary antibodies for one hour at room temperature. Blots were visualized using Immobilon™ Western Chemiluminescent HRP Substrate and the ChemiDoc™ XRS + imaging system (Bio-Rad). Image analysis and quantification were performed using Bio-Rad Image Lab software. The information of antibodies is detailed in Table S1.

### RNA extraction and qRT–PCR analysis

Total RNA was extracted using RNAiso plus (Takara) following the manufacturer’s protocol and subsequently reverse transcribed using the cDNA Reverse Transcription Kit. qRT–PCR reactions were performed with the SYBR Green PCR Kit and a ROCHE LightCycler 96 instrument. Expression data were normalized to β-actin mRNA levels and presented in arbitrary units calculated as 2^^Ct(β−actin−gene of interest)^. Primers used for qRT-PCR can be found in Table S3.

### Colony forming assay

The cells were seeded in a 6-well plate at a density of 500 cells per well in 2 mL of media. After a 2-week incubation period, the assays were terminated by fixing the cells in a methanol solution for 10 min, followed by staining with a 0.5% crystal violet solution for 30 min. The colony numbers were manually counted by a blinded observer.

### Flow cytometry-based assay

2 × 10^5^ cells were seeded overnight, collected, and labeled with 1 µM FerroOrange dye for 15–30 min. After centrifugation, cells were resuspended in PBS. Flow cytometry data (10,000 events) were acquired using a BD LSRFortessa™ Cell Analyzer and analyzed with FlowJo software.

2 million lung cancer cells were seeded overnight, exposed to Erastin, then stained with 1:1000 C11-BODIPY581/591. After digestion and collection, cells were resuspended in PBS. Fluorescent intensity was measured using BD LSRFortessa™, and lipid peroxidation was evaluated by analyzing FITC and PE channel ratios with FlowJo software.

### LC-MS/MS-based m^6^A level assay

Total RNA was isolated using RNAiso plus (Takara) following the manufacturer’s protocol. mRNA was subsequently extracted using a Dynabeads™ mRNA Purification Kit (Invitrogen) as previously described. For m^6^A quantification, 200 ng polyadenylated mRNAs were digested with 1.2 U of nuclease P1 (Sigma) dissolved in 25 µl of NH_4_OAc buffer (20 mM, pH = 5.3) at 42 °C for 2 h. Then, 3 µl of NH_4_HCO_3_ (1 M) and 1 U alkaline phosphatase (Sigma) were added, followed by an additional two hours of incubation at 37 °C. The samples were diluted with formic acid to 50 µl, filtered with a 0.22 µm syringe filter (Millipore), and 5 µl of the solution was injected into the LC-MS/MS. Nucleosides were separated by reversed-phase ultra-performance liquid chromatography on a C18 column, coupled to mass spectrometry detection with an Orbitrap Fusion™ Tribrid™ LC mass spectrometer (Thermo) in positive electrospray ionization mode. Nucleoside quantification was based on retention time and nucleoside-to-base ion transitions: 268-136 for A, and 282-150 for m^6^A. Using the standard curve generated from reference standards running in the same batch, the concentrations of A and m^6^A in the samples were calculated. The modification level of m^6^A was determined as the percentage of m^6^A out of the total amount of A, to normalize the amount of mRNA injected from different samples.

### LC-MS/MS-based GSH and GSSG detection

In the intricate LC-MS/MS-based sample preparation process, the initial steps involved the preparation of Solution 1 (comprising 100% LC-MS methanol) and Solution 2 (containing 25 mM ammonium acetate and 2.5 mM Na-Ascorbate in LC-MS water). Subsequently, Ellman’s reagent (20 mM) was meticulously added to Solution 2 in anticipation of subsequent experiments. During the sample collection phase, A549 cells were initially gathered at 4 °C and subjected to a brief centrifugation. Following this, 240 µL of Solution 1 and 60 µL of Solution 2 (containing Ellman’s Reagent) were judiciously added. After a brief vortexing and sonication, the samples underwent centrifugation, and the supernatant, rich in metabolites, was carefully transferred to ice and dried using a nitrogen desiccator. LC-MS analyses were promptly conducted on the same day as the extraction [60].

### ELISA assay

4-HNE and cysteine levels were measured using ELISA kits: the 4-HNE ELISA Kit (MEIMIAN, MM-13089H1) and the cysteine ELISA Kit (MyBioSource, MBS269913). Cell samples were lysed in RIPA buffer with protease and phosphatase inhibitors, then centrifuged at 12,000 rpm for 10 min to collect the supernatant. Levels of 4-HNE and cysteine in the lysates were measured following the manufacturer’s protocols and normalized to protein concentration using the BCA assay (KEYGEN, KGB2101).

### MDA measurement

Approximately 1 mL of pre-cooled extraction solution was added to prepare the cell lysate, followed by 30 cycles of sonication. The lysate was then centrifuged at 8000 g at 4 °C for 10 min, and the supernatant was collected. Test reagents were added according to the manufacturer’s instructions and incubated in a 100 °C water bath for 60 min. The mixture was then centrifuged at 10,000 g at room temperature for 10 min, and supernatant was used to detect absorbance at 532 and 600 nm using a microplate reader (BioTek).

### mRNA decay assay

The lung cancer cells were treated with actinomycin D at a final concentration of 5 μg/mL for the specified time intervals, after which total RNA was extracted. qRT-PCR was performed as described above. The mRNA half-life (t_1/2_) was computed using the formula ln 2/-slope, with GAPDH serving as the normalization reference.

### Dual luciferase assay

Fragments of either CBS-3′UTR-WT or CBS-3′UTR-Mut (where m^6^A was changed to T) were cloned into pmirGLO vectors to generate CBS-3′UTR-WT and CBS-3′UTR-Mut plasmids (Table S5). Luciferase activity was assessed using the Dual-Luciferase Reporter Assay System (Promega) 72 h post-transfection with CBS-3′UTR-WT or CBS-3′UTR-Mut. The relative Fluc/Rluc activity was determined by normalizing firefly luciferase activity to renilla luciferase activity.

### RNA-sequence

Paired-end reads were sequenced using Illumina NovaSeq 6000 with 150 bp read length. Quality control was firstly performed via Q30 calculations. After adapter trimming with Cutadapt(v 1.14)and low-quality read elimination with Trimmomatic(v 0.36), FastQC(v 0.11.5) software was used to do some quality control checks. Sequences were filtered to remove mitochondrial, ribosomal RNA and tRNA sequences with Bowtie2(v 2.2.9). Clean reads were aligned to Homo Sapiens genome (GENCODE Release 36, GRCh38.p13) by STAR(v 2.4.0). Reads were counted using featureCounts(v 1.6.3). DESeq2(v 1.42.0) was used for differential gene expression analysis. (*p*-value < 0.05 and |Fold change | > 1.5).

### Metabolome analysis

Beginning with the careful removal of A549 cell culture medium and a subsequent PBS wash, the extraction process employed 800 μL of cold methanol/acetonitrile/water (2:2:1,v/v/v). The resulting mixture underwent centrifugation at 14000 g for 5 min, and the supernatant was judiciously collected and subjected to drying in a vacuum centrifuge. For LC-MS analysis, the dried samples were re-dissolved in 100 μL acetonitrile/water (1:1, v/v) solvent. Maintaining analytical stability and repeatability, quality control (QC) samples were meticulously prepared by pooling 10 μL from each sample, systematically inserted for analysis every 5 samples.

The LC-MS analysis employed state-of-the-art equipment, including an UHPLC (1290 Infinity LC, Agilent Technologies) coupled with a quadrupole time-of-flight (AB Sciex TripleTOF 6600) at Shanghai Applied Protein Technology Co., Ltd.Utilizing both HILIC and RPLC separation techniques, chromatographic separation ensured robust performance in both ESI positive and negative modes. The ESI source conditions were set as follows: Ion Source Gas1 (Gas1) as 60, Ion Source Gas2 (Gas2) as 60, curtain gas (CUR) as 30, source temperature: 600°C, IonSpray Voltage Floating (ISVF) ± 5500 V. In MS only acquisition, the instrument was set to acquire over the m/z range 60-1000 Da, and the accumulation time for TOF MS scan was set at 0.20 s/spectra. In auto MS/MS acquisition, the instrument was set to acquire over the m/z range 25-1000 Da, and the accumulation time for product ion scan was set at 0.05 s/spectra. The product ion scan is acquired using information dependent acquisition (IDA) with high sensitivity mode selected. The parameters were set as follows: the collision energy (CE) was fixed at 35 V with ± 15 eV; declustering potential (DP), 60 V (+) and −60 V (−); exclude isotopes within 4 Da, candidate ions to monitor per cycle: 10.

The raw MS data underwent thorough processing using ProteoWizard MS Convert before importing into freely available XCMS software. For peak picking, the following parameters were used: centWave m/z = 10 ppm, peakwidth = c (10, 60), prefilter = c (10, 100). For peak grouping, bw = 5, mzwid = 0.025, minfrac = 0.5 were used. CAMERA (Collection of Algorithms of MEtabolite pRofile Annotation) was sued for annotation of isotopes and adducts. In the extracted ion features, only the variables having more than 50% of the nonzero measurement values in at least one group were kept. Compound identification of metabolites was performed by comparing of accuracy m/z value (<10 ppm), and MS/MS spectra with an in-house database established with available authentic standards.The processed data were then subjected to multivariate data analysis using the R package (ropls), including Principal Component Analysis (PCA) and Orthogonal Partial Least-Squares Discriminant Analysis (OPLS-DA). To ensure the robustness of the model, a 7-fold cross-validation and response permutation testing were conducted. Statistical significance was assessed through Student’s t-test, with a stringent threshold of *p* value < 0.05 indicating significant changes.

### MeRIP-sequence

MeRIP-seq assay was performed by Cloudseq Biotech Inc. In brief, m^6^A RNA immunoprecipitation (IP) was carried out utilizing a GenSeq™ m^6^A RNA IP Kit (GenSeq). Both the input and m^6^A IP samples were prepared for NGS. The library was constructed using a NEBNext® Ultra II Directional RNA Library Prep Kit (NEB). The library’s quality was assessed using a BioAnalyzer 2100 system (Agilent).

For MeRIP-seq analysis: Paired-end reads were sequenced on the Illumina NovaSeq 6000 platform with a 150 bp read length. Quality control was initiated by calculating Q30 scores. Subsequently, adapter trimming was performed using Cutadapt (v1.14), and low-quality reads were removed with Trimmomatic (v0.36). Quality checks were conducted using FastQC (v0.11.5) software. To eliminate mitochondrial, ribosomal RNA, and tRNA sequences, Bowtie2 (v2.2.9) was employed. Clean reads were then aligned to Homo sapiens transcripts (GENCODE Release 36, GRCh38.p13) using Bowtie2 (v2.2.9) with the parameters (–local -x). Methylated sites on peaks were identified using MACS2 (v2.2.7.1) peak-calling software, using corresponding input samples as controls. Differentially methylated sites were determined with the BEDTools map function in the R language. For peak annotation, the R package ChIPseeker (v1.38.0) [61] was utilized. The distribution of m^6^A peaks on mRNA features was visualized using the R package Guitar (v2.18.0). Enriched motifs within m^6^A peaks were identified with HOMER [62].

### Whole exome sequencing of PDOs and matched primary lung tumor tissues

Genomic DNA was extracted from patient-derived tumor organoids (PDOs) and matched primary lung tissues for whole-exome sequencing. Libraries were prepared using the Agilent SureSelect Human All Exon V6 kit (64 Mb) and sequenced on the Illumina NovaSeq X platform with 150 bp paired-end reads, achieving an average coverage depth of over 100×. Raw sequencing reads were subjected to quality control using fastp (v1.0.1) [63], which automatically trimmed adapter sequences and filtered out low-quality bases at read ends. Quality reports were generated as part of this process. Clean reads were then aligned to the human reference genome (hg19) using BWA (v0.7.19). The resulting BAM files were sorted and indexed with Samtools, and duplicate reads were removed [64]. Base quality score recalibration (BQSR) was performed using GATK (v4.6.2.0). High-quality BAM files were processed with GATK HaplotypeCaller for variant calling, with single nucleotide polymorphisms (SNPs) and insertions/deletions (INDELs) filtered based on GATK best practice recommendations. Somatic mutations were identified using GATK Mutect2, and high-confidence calls were obtained through population database filtering and stringent post-processing criteria. All variants were functionally annotated with ANNOVAR (2025-06), incorporating multiple public databases, including RefSeq, ClinVar, COSMIC, the 1000 Genomes Project, and gnomAD. Copy number variation (CNV) analysis was performed in tumor-only mode using CNVkit (v0.9.12) with a pre-built flat reference. Statistical analyses and data visualization were conducted in Python 3.10 using packages such as Pandas, Matplotlib, and Seaborn.

### Survival analysis and risk score model construction

To assess the prognostic significance of candidate genes in lung adenocarcinoma (LUAD) patients, clinical survival data—including overall survival (OS) and progression-free interval (PFI)—were retrieved from the TCGA-LUAD cohort. Kaplan–Meier survival curves were generated to compare outcomes between high and low gene expression groups, with statistical significance evaluated using the log-rank test. To capture the combined prognostic impact of multiple genes, a multigene risk score model was constructed based on the Cox proportional hazards regression. Gene expression levels served as independent variables in the multivariate Cox model, and a risk score for each patient was calculated using the following formula:

$${{\rm{Risk\; Score}}}_{i}=\mathop{\sum }\limits_{j=1}^{n}{\beta }_{j}\times {{\rm{Exp}}}_{{ji}}$$

Where βj is the regression coefficient for gene j, and Expji represents the expression level of gene j in patient i. Patients were then stratified into high-risk and low-risk groups according to the median risk score. Kaplan–Meier analysis and the log-rank test were performed to evaluate survival differences between the two groups. All analyses and visualizations were carried out using the survival and survminer packages in R.

### Statistical analysis

To determine statistical significance, comparisons between two groups were performed using an unpaired Student’s t-test, comparisons between multiple groups were performed using a one-way ANOVA, comparisons between two or multiple groups with additional treatment conditions or repeated measurements at different time periods were performed using a two-way ANOVA, and detailed statistical analyses of high-throughput sequencing are described in the “Materials and Methods” section. For additional information on statistical analyses, please refer to the figure legends.

## Supplementary information

Figure S1

Figure S2

Figure S3

Figure S4

Figure S5

Figure S6

Figure S7

Figure S8

Figure S9

Figure S10

Figure S11

Figure S12

Supplementary information

original data-ddx3x

## Data Availability

All MeRIP-seq datasets have been deposited in the NCBI’s Gene Expression Omnibus (GEO) and are publicly accessible under accession number GSE262058.All RNA-seq datasets have also been deposited in the NCBI’s Gene Expression Omnibus (GEO) and can be accessed using accession number GSE262680.All proteomic datasets have been deposited in the iProX database and are publicly available under accession number PXD051043.All metabolomic datasets have been deposited in the MetaboLights database and are accessible under accession number MTBLS9747. All whole exome sequencing raw data were deposited in NCBI Sequence Read Archive (SRA) and are accessible through project numbers PRJNA1283526. Any additional information required to reanalyze the data reported in this work paper is available from the Lead Contact upon request.
