# Supplementary figures and images for: Targeting DDX3X suppresses progression of KRAS-driven lung cancer by disrupting antioxidative homeostasis and inducing ferroptosis
Source: Cell Death Dis. 2025 Aug 30;16(1):660. doi: 10.1038/s41419-025-07980-8 (PMC12398621; doi:10.1038/s41419-025-07980-8)

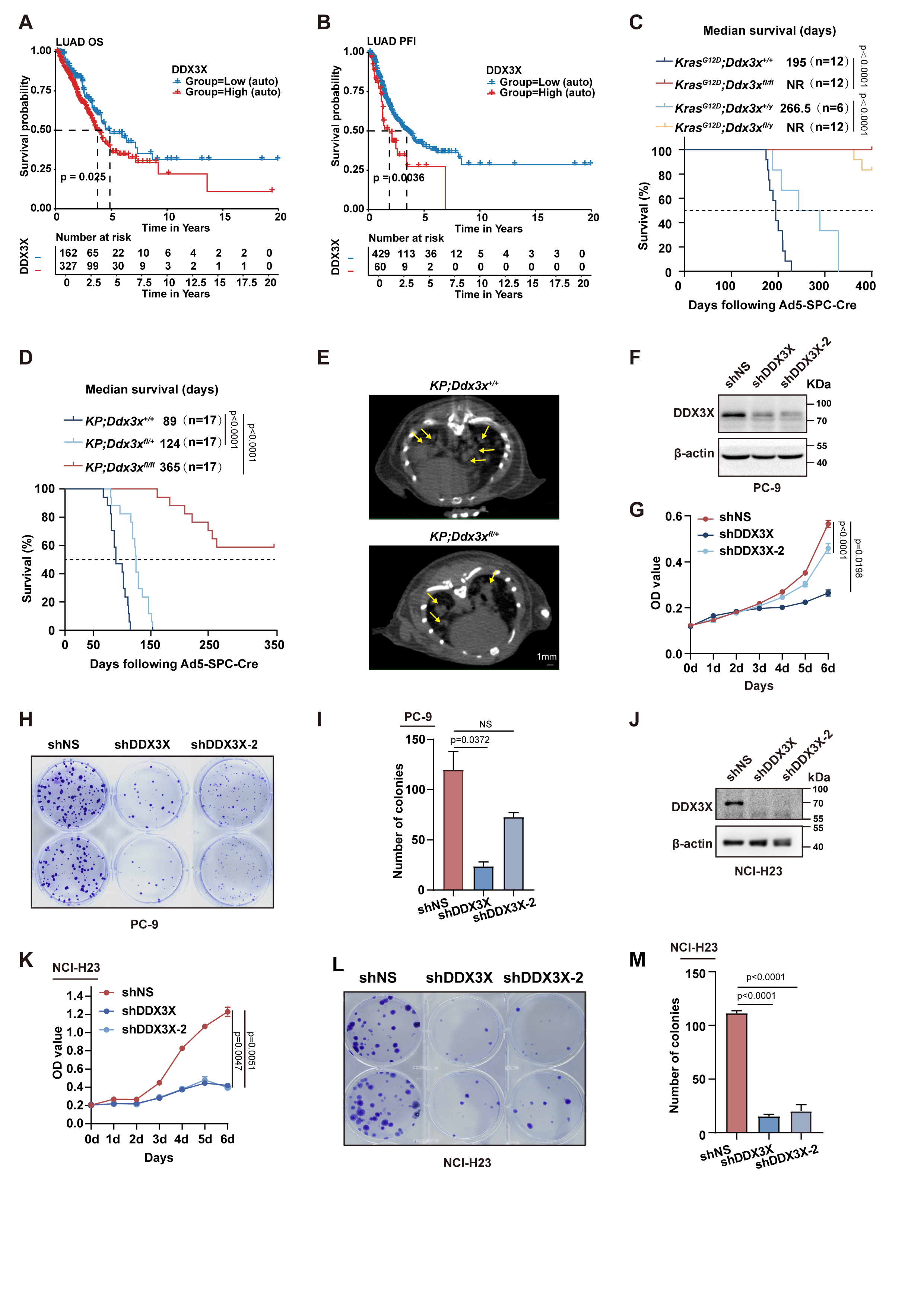

Supplement: Supplementary file 1 — Figure S1 [file 41419_2025_7980_MOESM1_ESM.tif]

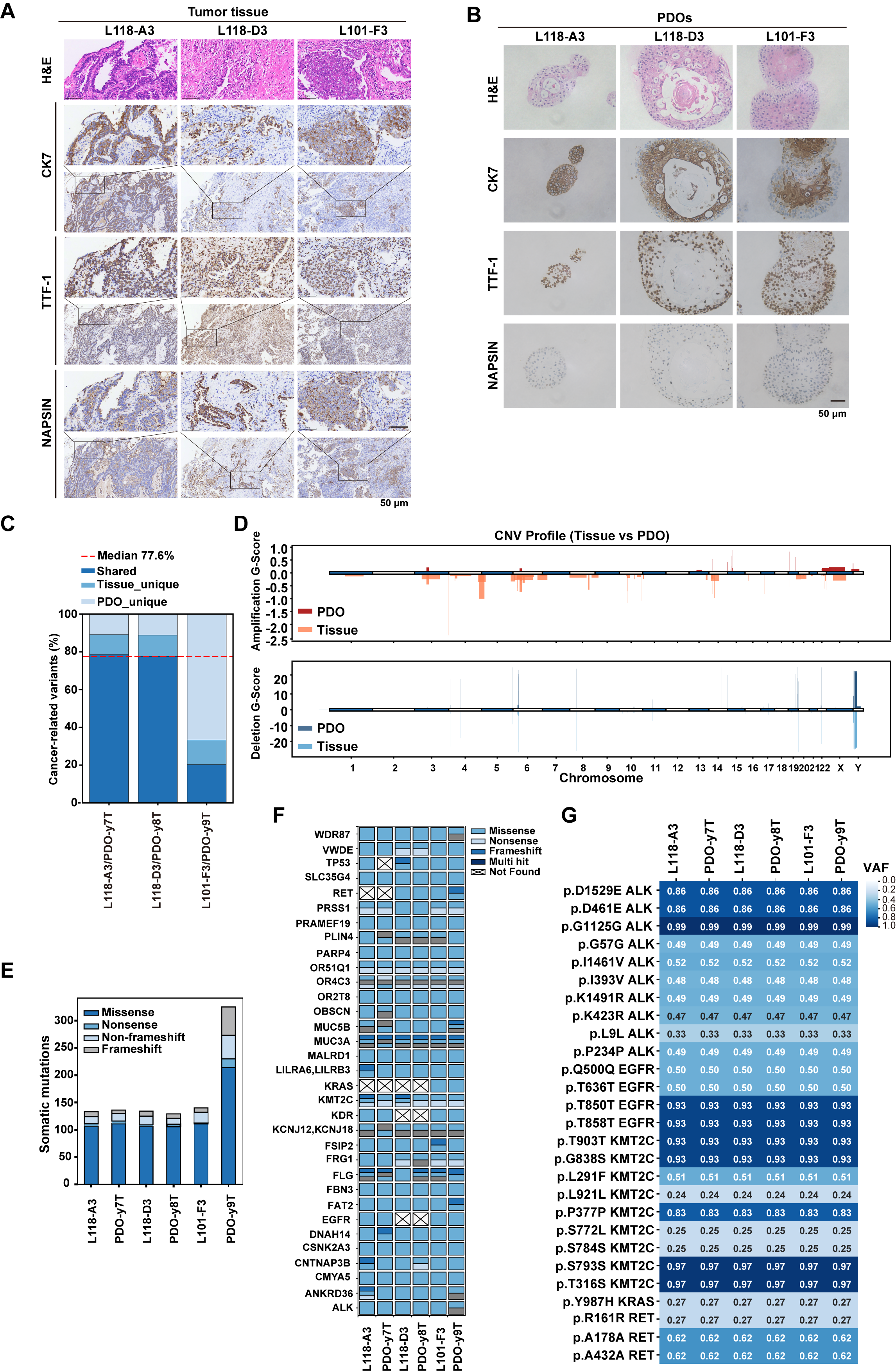

Supplement: Supplementary file 2 — Figure S2 [file 41419_2025_7980_MOESM2_ESM.tif]

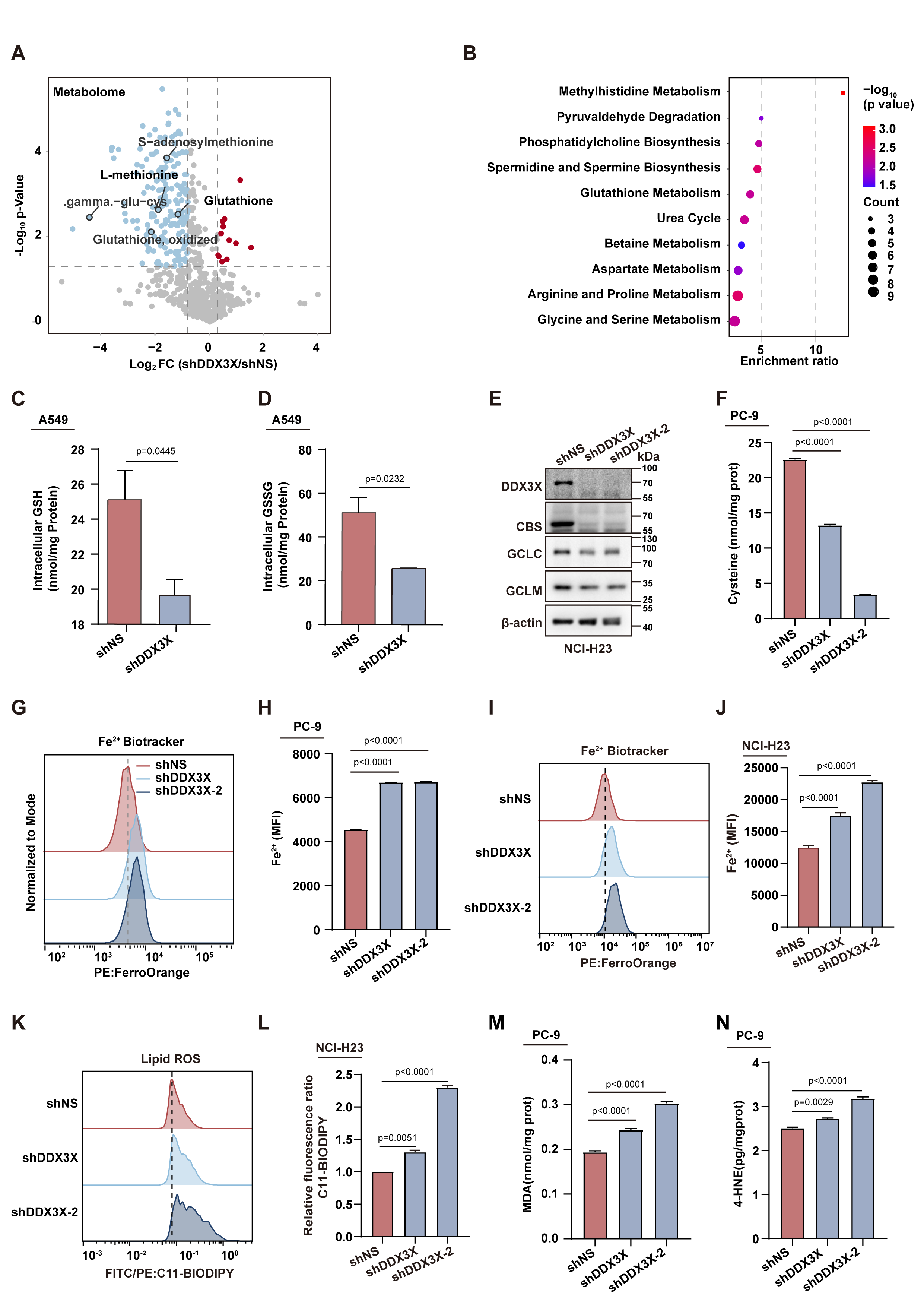

Supplement: Supplementary file 3 — Figure S3 [file 41419_2025_7980_MOESM3_ESM.tif]

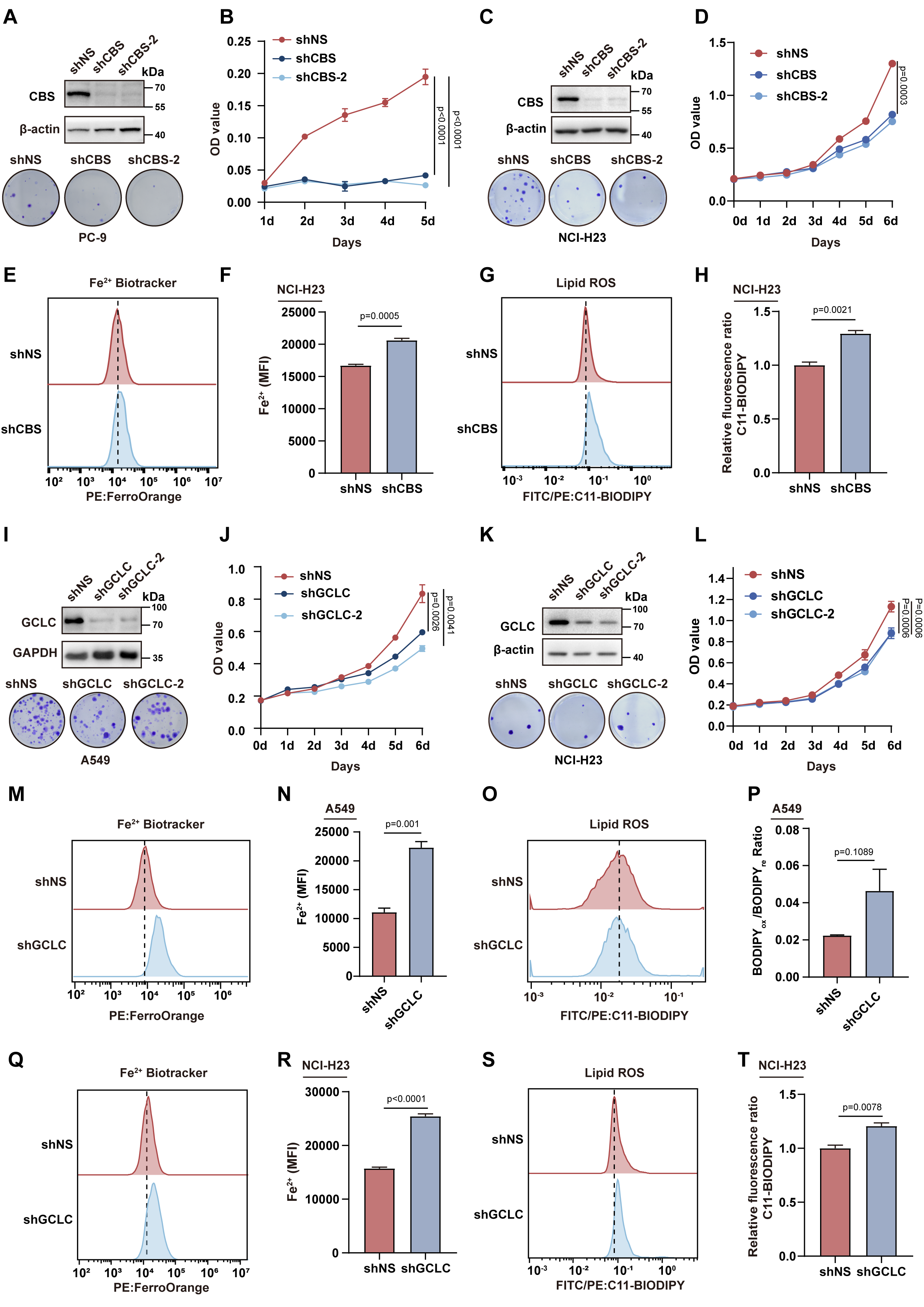

Supplement: Supplementary file 4 — Figure S4 [file 41419_2025_7980_MOESM4_ESM.tif]

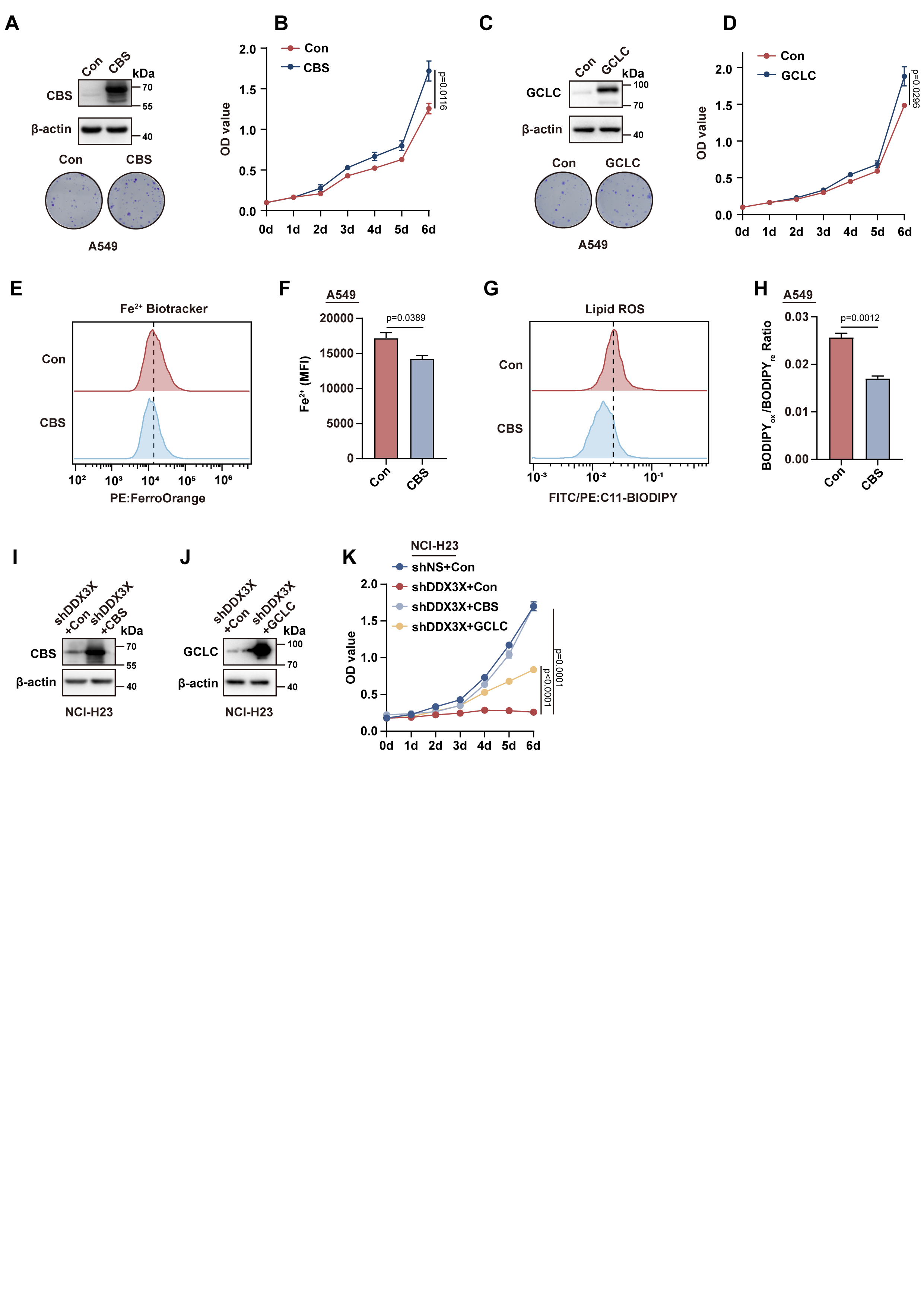

Supplement: Supplementary file 5 — Figure S5 [file 41419_2025_7980_MOESM5_ESM.tif]

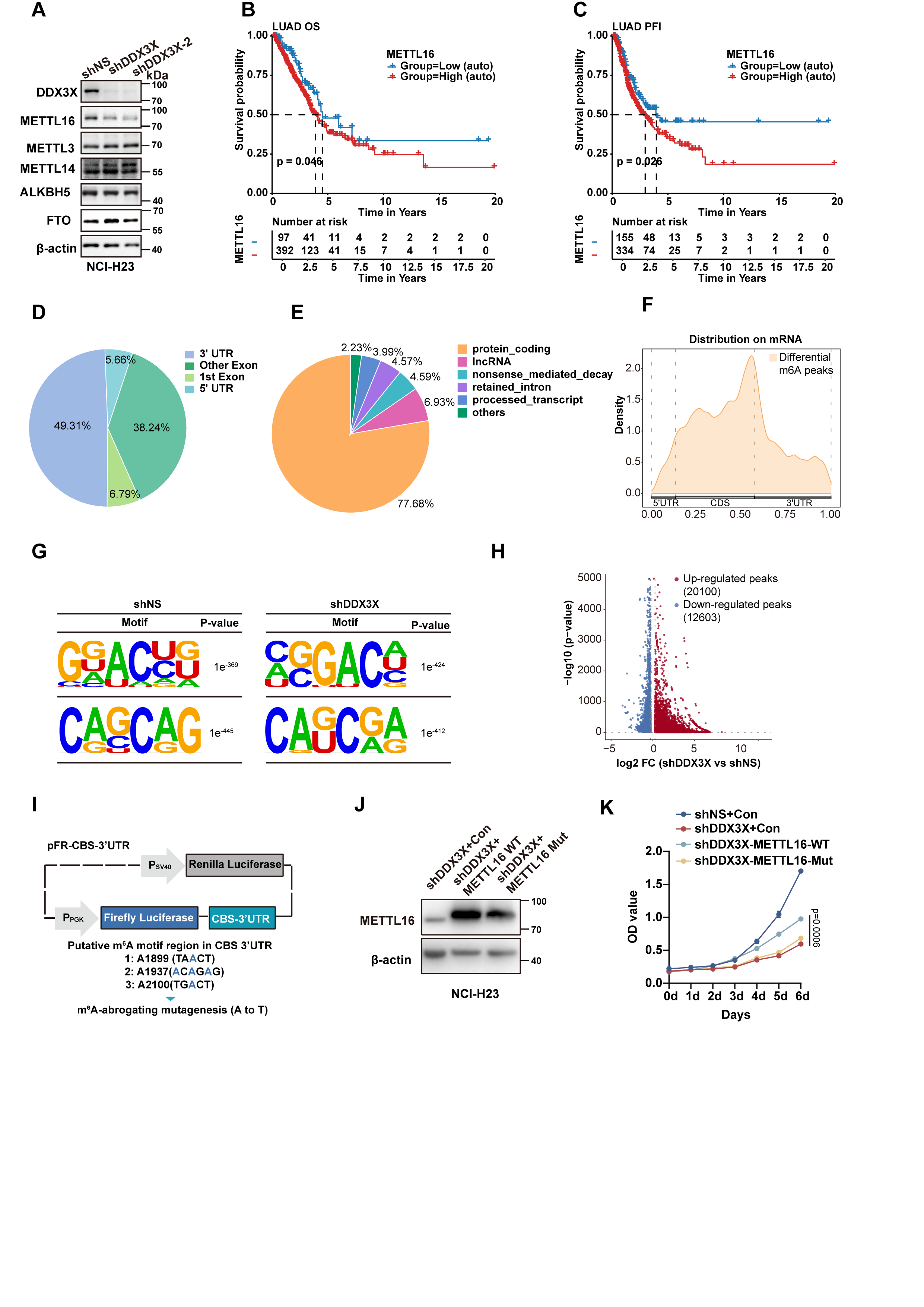

Supplement: Supplementary file 6 — Figure S6 [file 41419_2025_7980_MOESM6_ESM.tif]

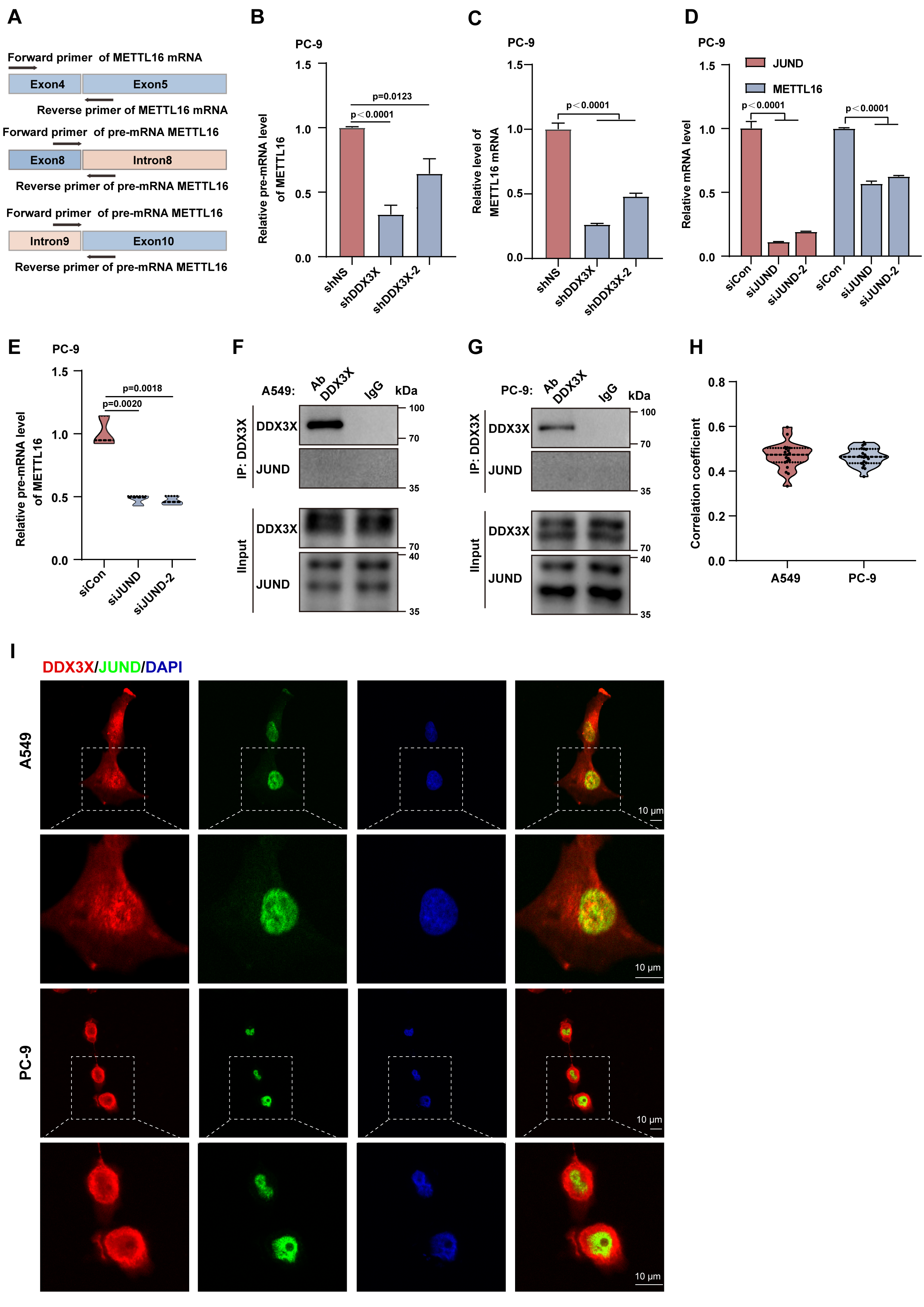

Supplement: Supplementary file 7 — Figure S7 [file 41419_2025_7980_MOESM7_ESM.tif]

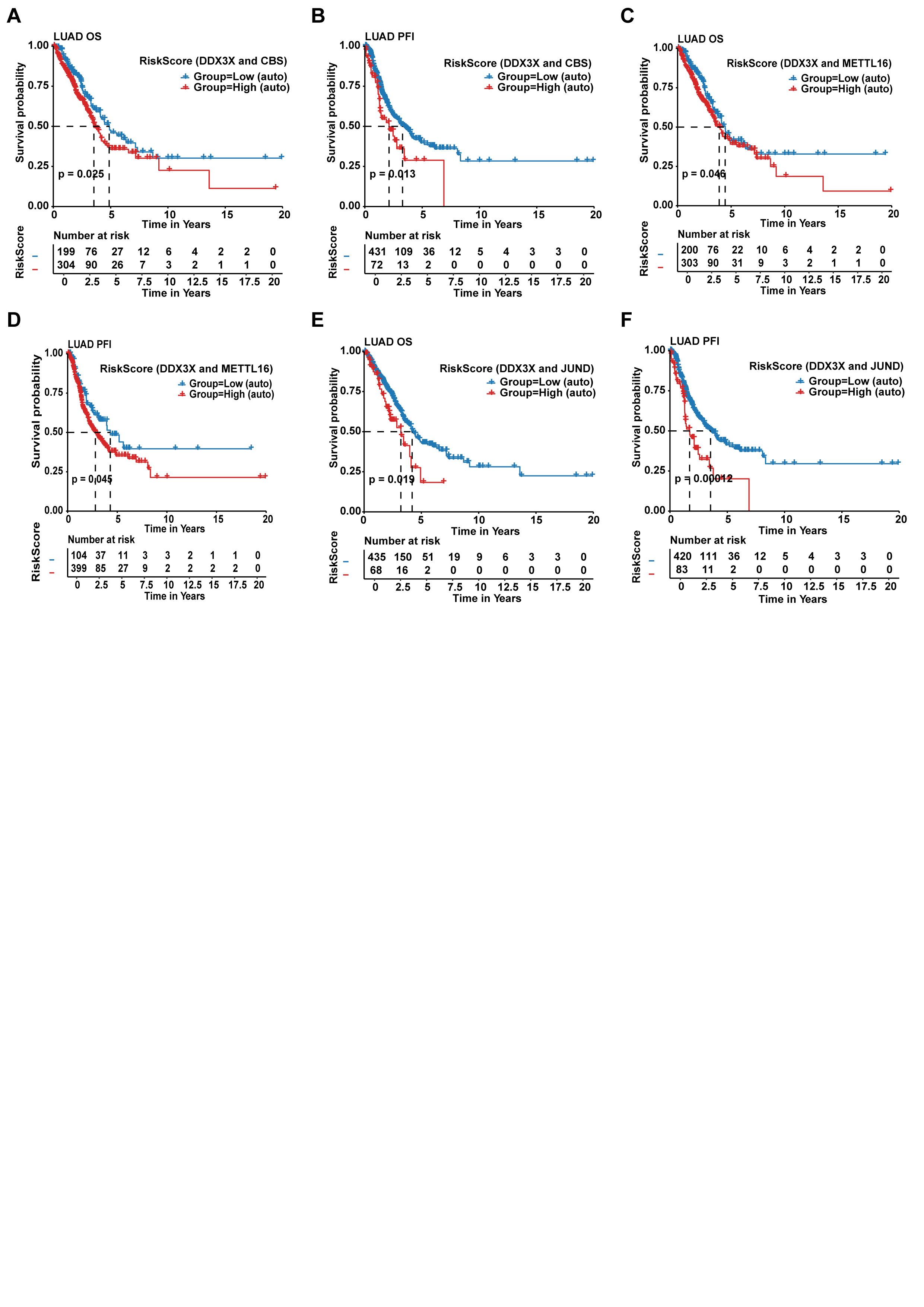

Supplement: Supplementary file 8 — Figure S8 [file 41419_2025_7980_MOESM8_ESM.tif]

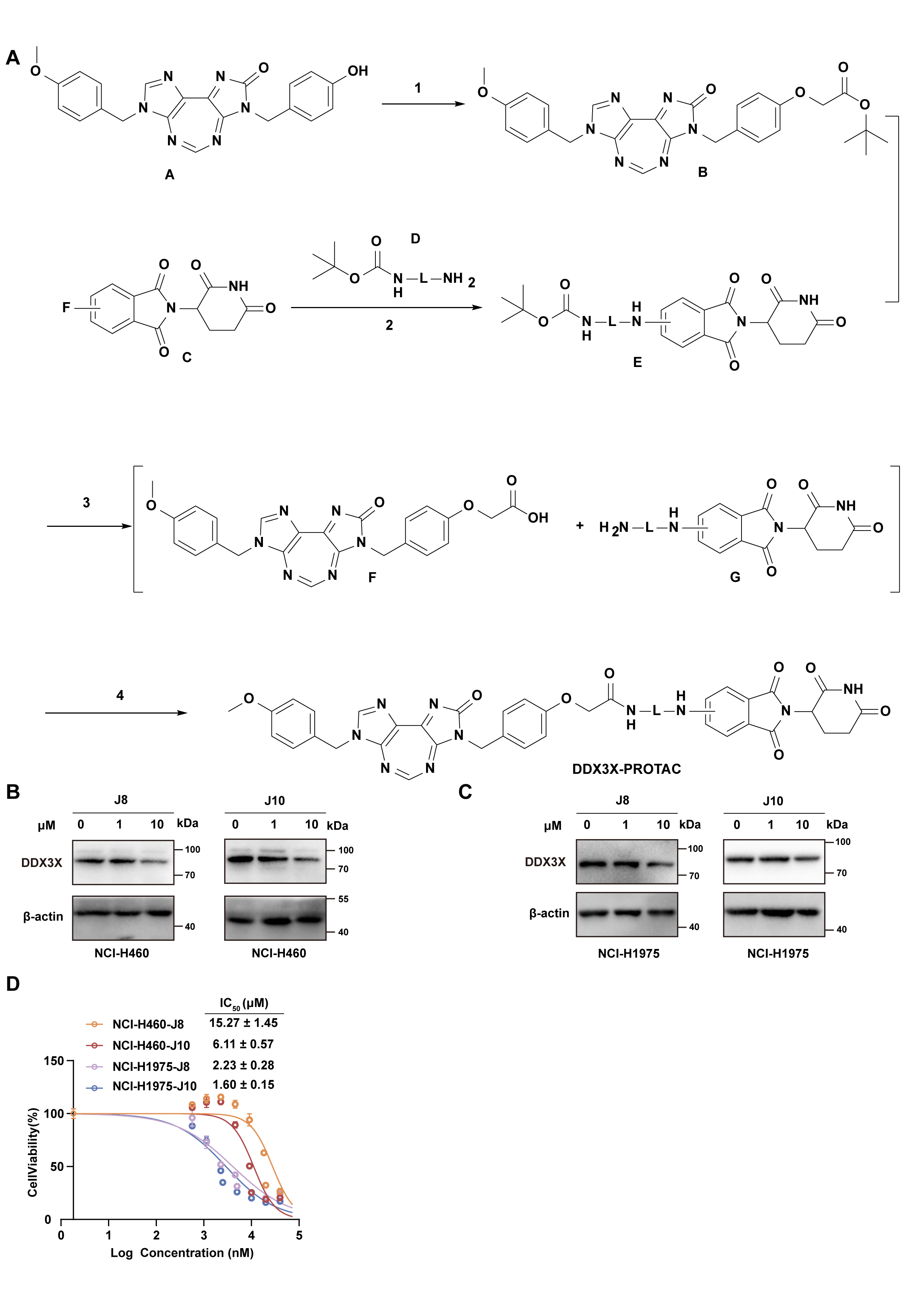

Supplement: Supplementary file 9 — Figure S9 [file 41419_2025_7980_MOESM9_ESM.tif]

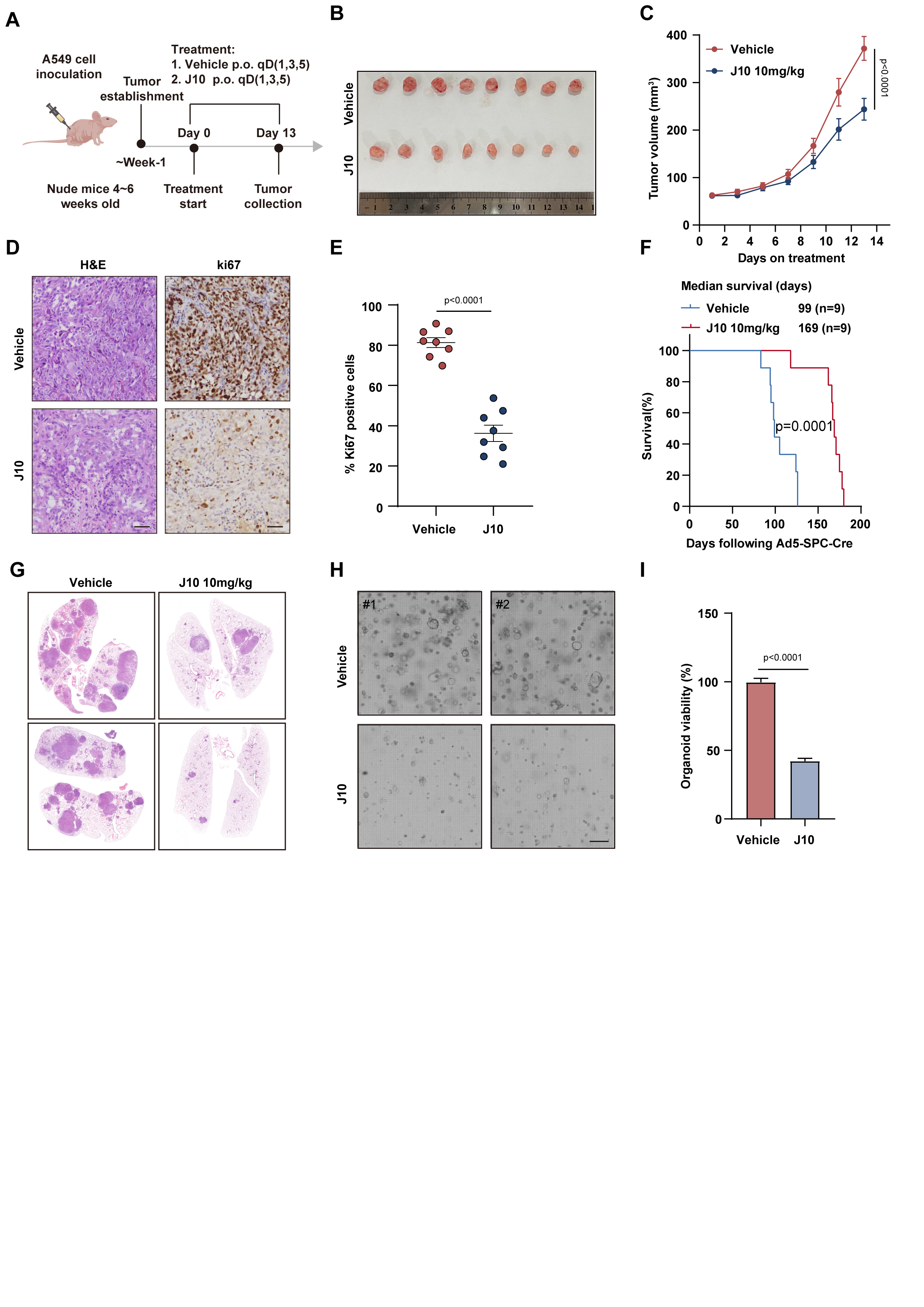

Supplement: Supplementary file 10 — Figure S10 [file 41419_2025_7980_MOESM10_ESM.tif]

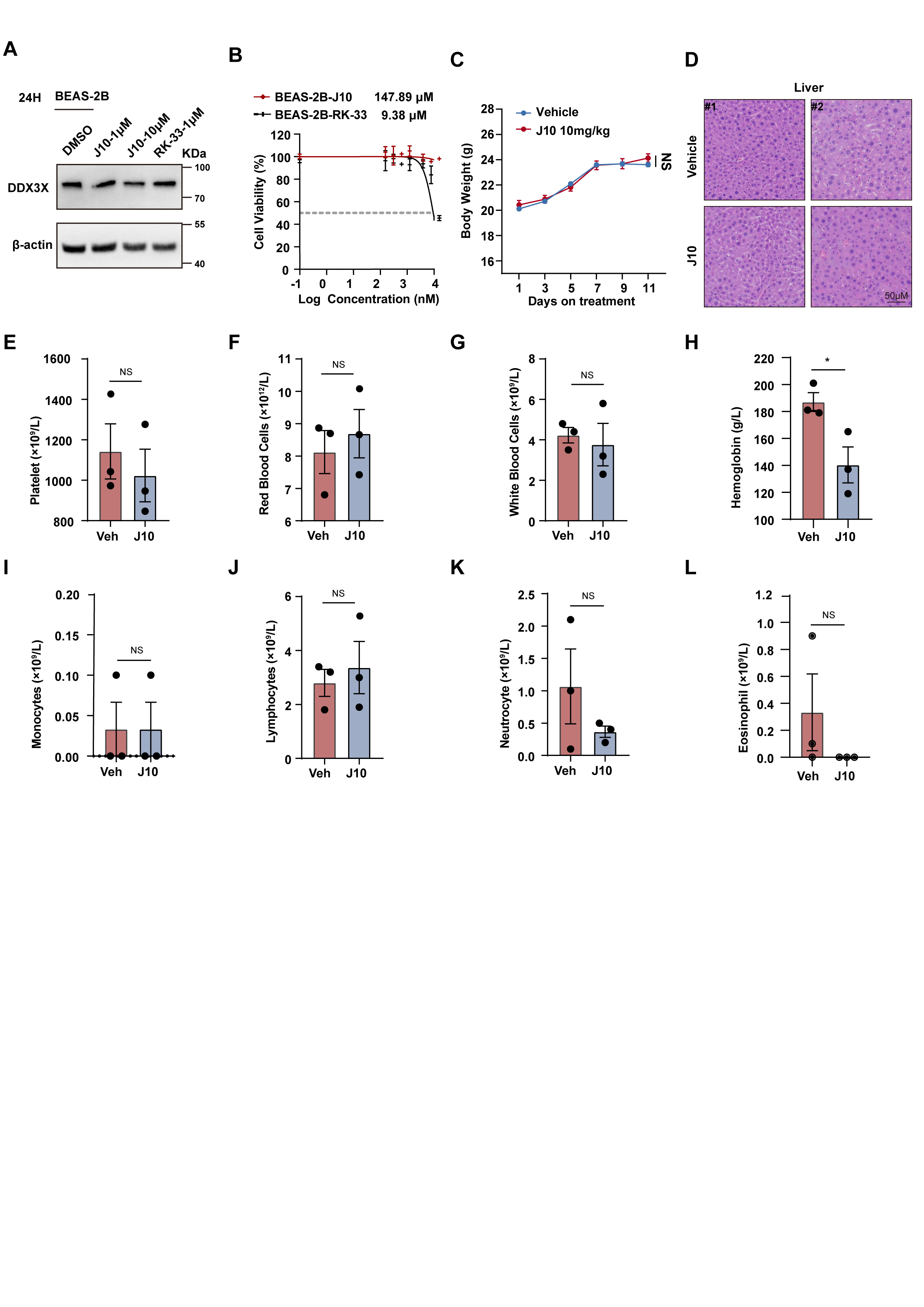

Supplement: Supplementary file 11 — Figure S11 [file 41419_2025_7980_MOESM11_ESM.tif]

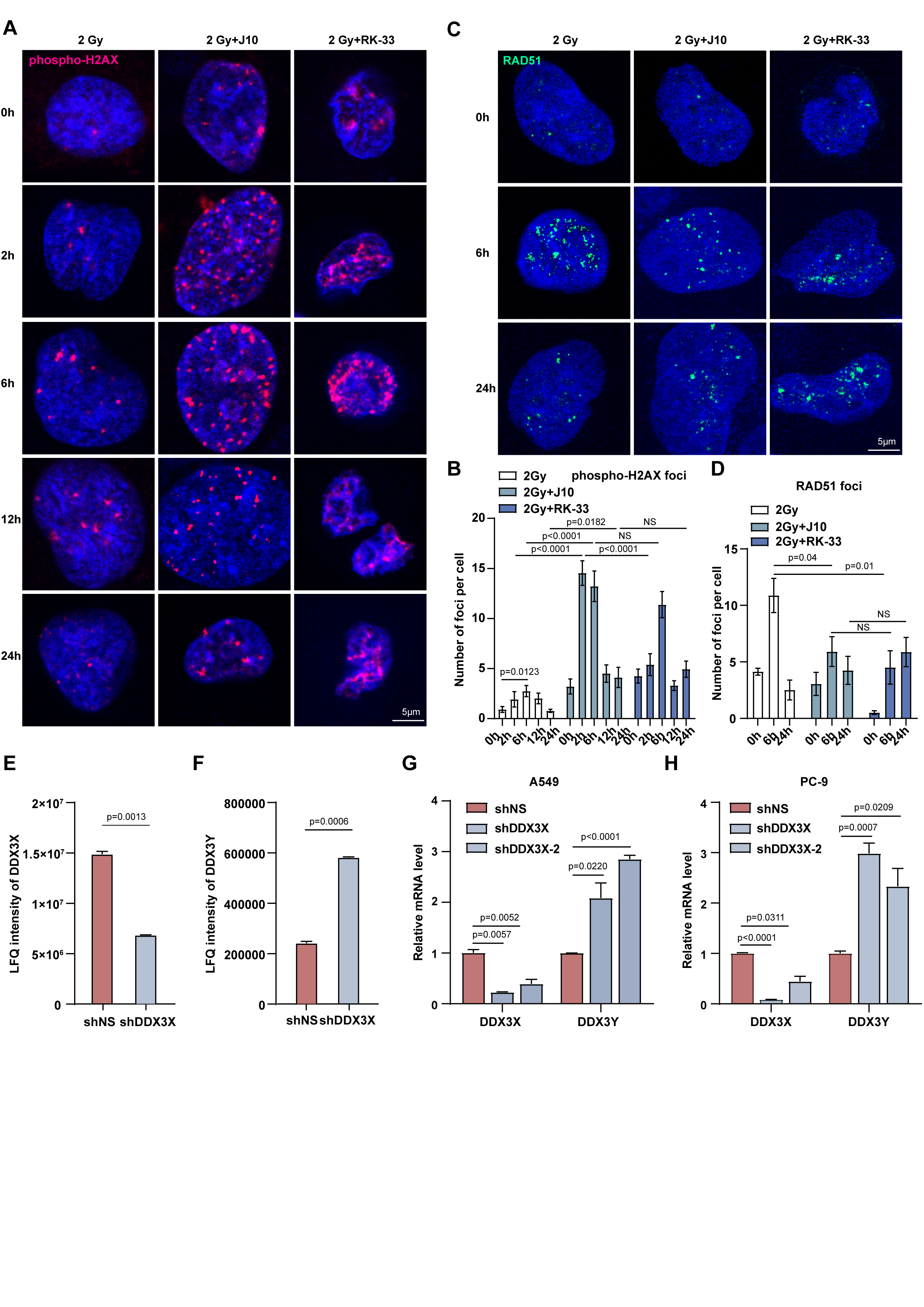

Supplement: Supplementary file 12 — Figure S12 [file 41419_2025_7980_MOESM12_ESM.tif]
