# Supplementary material for: Targeting DDX3X suppresses progression of KRAS-driven lung cancer by disrupting antioxidative homeostasis and inducing ferroptosis
Source: Cell Death Dis. 2025 Aug 30;16(1):660. doi: 10.1038/s41419-025-07980-8 (PMC12398621; doi:10.1038/s41419-025-07980-8)
Supplement: Supplementary file 13 — Supplementary information [file 41419_2025_7980_MOESM13_ESM.docx]

**Supplementary figure legends**

**Figure S1. Inhibition of DDX3X prolongs survival of *Kras^G12D^* driven lung cancer mice and suppresses lung cancer cells proliferation.**

**(A and B)** Kaplan-Meier curves for **(A)** overall survival (OS) and **(B)** progression-free interval (PFI) between patients with high versus low DDX3X mRNA expression in the TCGA lung adenocarcinoma cohort. (**C)** Kaplan-Meier curves showing the overall survival of mice bearing lung cancer distinguished by their genetic backgrounds: *Kras^G12D^; Ddx3x^+/+^*(n=12), *Kras^G12D^;Ddx3x^fl/fl^*(n=12), *Kras^G12D^;Ddx3x^+/y^* (n=6), and *Kras^G12D^;Ddx3x^fl/y^* (n=12) (****p < 0.0001, Log-rank (Mantel-Cox) test). **(D)** Kaplan-Meier curves showing the overall survival of mice with lung cancer distinguished by their genetic backgrounds: *KP;Ddx3x^+/+^* (n=17), *KP;Ddx3x^fl/+^* (n=17), and *KP;Ddx3x^fl/fl^* (n=17) (Log-rank (Mantel-Cox) test). **(E)** Representative transverse micro-CT images of wild-type (WT) and DDX3X knockout (KO) mice were captured at uniform endpoint event time points. The selected images correspond to the location of the largest tumour diameter. Tumors are marked with yellow arrows. Scale bar: 1mm. **(F)** Immunoblot assay of DDX3X in DDX3X shRNA transfected PC-9 cells. **(G)** A 6-day period cell proliferation assay of shNS or shDDX3X PC-9 cells (n=3, mean ± SEM, Two-way ANOVA with Dunnett's post hoc test). **(H)** Clonogenic assay of PC-9 cells transduced with shNS or shDDX3X, the colonies were stained and assessed after a 2-week incubation period. **(I)** Quantification of colony formation in PC-9 cells transduced with shNS or shDDX3X. (n=3, mean ± SEM, unpaired Student’s t test). **(J)** Immunoblot assay of DDX3X in DDX3X shRNA transfected NCI-H23 cells (n=3). **(K)** A 6-day period cell proliferation assay of shNS or shDDX3X NCI-H23cells (n=3, mean ± SEM, Two-way ANOVA with Dunnett's post hoc test). **(L)** Clonogenic assay of NCI-H23 cells transduced with shNS or shDDX3X, the colonies were stained and assessed after a 2-week incubation period. **(M)** Quantification of colony formation in NCI-H23 cells transduced with shNS or shDDX3X (n=3, mean ± SEM, unpaired Student’s t test).

**Figure S2. Histomorphological and genomic characterization of patient-derived tumor organoid (PDOs) models.**

**(A and B)** Similarities in histomorphology (hematoxylin and eosin) and pathological marker expression (immunohistochemistry) between primary tumor tissues **(A)** and matched PDOs **(B)**. Representative images of LUAC-specific markers [cytokeratin 7 (CK7), thyroid transcription factor (TTF1), and napsin-A in patient-matched tumor tissue and PDOs. scale bar 50 μm. **(C)** Concordance of cancer-related variants between tumor tissue and organoids. Median concordance is indicated. **(D)** Genome-wide comparison of copy number alterations (CNAs) between tumor tissue and organoids. Copy number gain (upper panel) and loss (bottom panel) peaks were plotted separately. **(E)**Total number of cancer-relevant somatic mutations identified in tumor tissue and organoids (somatic mutations were identified using COSMIC database). **(F)** Heatmap showing somatic mutations in oncogenes and tumor suppressor genes identified in tumor tissue and organoids. **(G)** Heatmap comparing mutation VAFs of COSMIC cancer genes between tumor tissue and PDOs for three representative lung cancer patients. VAF, variant allele frequency.

**Figure S3. Metabolic reprogramming in DDX3X deficient lung cancer cells.**

**(A)** Volcano plot showing the fold change and variance for biochemicals, providing a comprehensive overview of the differential regulation of metabolites. Differentially expressed biochemicals (DEB) are denoted by red and blue points, indicating up-regulated (fold change>1.23 and *p*-Value<0.05) and down-regulated (fold change<0.57 and *p*-Value<0.05) changes, respectively. **(B)** Scatterplot showing the prominent KEGG metabolism pathways among differentially regulated biochemicals (DEB). Point size and color reflect the DEB hit count (number displayed) and *p* value, respectively. The top ten KEGG pathways are ranked by enrichment ratio (X axis). **(C and D)** Intracellular levels of reduced glutathione (GSH) **(C)** and oxidized glutathione (GSSG) **(D)** in shNS or shDDX3X A549 cells determined by LC-MS (n=3, mean ± SEM, *p < 0.05, unpaired Student’s t test). **(E)** Immunoblot analysis of DDX3X, CBS, GCLC, and GCLM in NCI-H23 cells. β-actin was employed as a loading control. **(F)** Measurement of cysteine levels in PC-9 shDDX3X or shNS cells. n = 3, mean ± SEM with ordinary One-way ANOVA. **(G)** Flow cytometry assay of Ferrous ion (Fe^2+^) fluorescence in PC-9 cells transduced with lentiviral shNS or shDDX3X. **(H)** Quantified mean fluorescence intensity (MFI) in **(G)** (n=3, mean ± SEM, One-way ANOVA and Tukey's post hoc tests). **(I)** Flow cytometry assay of Ferrous ion (Fe^2+^) fluorescence in NCI-H23 cells transduced with lentiviral shNS or shDDX3X. **(J)** Quantification of mean fluorescence intensity (MFI) in **(I)** (n=3, mean ± SEM, One-way ANOVA). **(K)** Flow cytometry analysis assessed lipid peroxidation through C11-BODIPY fluorescence in shNS or shDDX3X NCI-H23 cells. FITC-BODIPY C11 and PE-BODIPY C11 represent oxidized (ox) and reduced (re) populations, respectively. **(L)** Quantification of the ratio of oxidized (ox) to reduction (re) fluorescence intensity in **(K)** (n=3, mean ± SEM, One-way ANOVA). (**M and N)** Measurement of malondialdehyde (MDA) **(M)**, and 4-hydroxynonenal (4-HNE) **(N)** levels in PC-9 shDDX3X or shNS cells. n = 3, mean ± SEM with ordinary One-way ANOVA.

**Figure S4. CBS and GCLC are required for lung cancer cells’ proliferation.**

**(A)** CBS protein levels in PC-9 cells transfected with non-targeting shRNA (shNS) or CBS-targeting shRNA (shCBS) (top), and representative images of colony formation in shNS or shCBS PC-9 cells (bottom) (n=3). **(B)** A 5-day period cell proliferation assay in shNS or shCBS PC-9 cells (n=3, mean ± SEM, Two-way ANOVA with Dunnett's post hoc test). **(C)** CBS protein levels in NCI-H23 cells transfected with non-targeting shRNA (shNS) or CBS-targeting shRNA (shCBS) (top), and representative images of colony formation in shNS or shCBS NCI-H23 cells (bottom) (n=3). **(D)** A 6-day period cell proliferation assay in shNS or shCBS NCI-H23 cells (n=3, mean ± SEM, Two-way ANOVA with Dunnett's post hoc test). **(E)** FACs analysis of ferrous ion (Fe^2+^) levels in CBS knockdown NCI-H23 cells. **(F)** Quantification of the mean fluorescence intensity (MFI) of ferrous ions (Fe^2+^) in **(E)** (n=3, mean ± SEM, One-way ANOVA). **(G)** Assessment of lipid peroxidation via C11-BODIPY 581/591 staining in control or CBS knockdown NCI-H23 cells. **(H)** Quantification of C11-BODIPY fluorescence intensity in the specified cells in **(G)**. **(I)** GCLC protein levels in A549 cells transfected with non-targeting shRNA (shNS) or GCLC-targeting shRNA(shGCLC) (top), and representative images of colony formation in shNS or shGCLC A549 cells (bottom) (n=3). **(J)** A 6-day period cell proliferation assay in shNS or shGCLC A549 cells (n=3, mean ± SEM, Two-way ANOVA with Dunnett's post hoc test). **(K)** GCLC protein levels in NCI-H23 cells transfected with non-targeting shRNA (shNS) or GCLC-targeting shRNA(shGCLC) (top), and representative images of colony formation in shNS or shGCLC NCI-H23 cells (bottom) (n=3). **(L)** A 6-day period cell proliferation assay in shNS or shGCLC NCI-H23 cells (n=3, mean ± SEM, Two-way ANOVA with Dunnett's post hoc test). **(M)** FACs analysis of ferrous ion (Fe^2+^) levels in GCLC knockdown A549 cells. **(N)** Quantification of the mean fluorescence intensity (MFI) of ferrous ions (Fe^2+^) in **(M)** (n=3, mean ± SEM, One-way ANOVA). **(O)** Assessment of lipid peroxidation via C11-BODIPY 581/591 staining in control or GCLC knockdown A549 cells. **(P)** Quantification of C11-BODIPY fluorescence intensity in the specified cells in **(O)**. **(Q)** FACs analysis of ferrous ion (Fe^2+^) levels in GCLC knockdown NCI-H23 cells. **(R)** Quantification of the mean fluorescence intensity (MFI) of ferrous ions (Fe^2+^) in **(Q)** (n=3, mean ± SEM, One-way ANOVA). **(S)** Assessment of lipid peroxidation via C11-BODIPY 581/591 staining in control or GCLC knockdown NCI-H23 cells. **(T)** Quantification of C11-BODIPY fluorescence intensity in the specified cells in **(S)**.

**Figure S5. CBS or GCLC regulates ferroptosis.**

**(A)** CBS protein levels in A549 cells transfected with non-targeting vector (Control) or overexpressing CBS plasmids (OE CBS) (top). Representative images depicting colony formation in control or OE CBS A549 cells (bottom). **(B)** A 6-day period cell proliferation analysis of control or OE CBS A549 cells (n=3, mean ± SEM, Two-way ANOVA with Dunnett's post hoc test). **(C)** GCLC protein levels in A549 cells transfected with non-targeting vector (Control) or overexpressing GCLC plasmids (OE GCLC) (top). Representative images of colony formation in control or OE GCLC A549 cells (bottom). **(D)** A 6-day period cell proliferation analysis of control or OE GCLC-A549 cells (n=3, mean ± SEM, Two-way ANOVA with Dunnett's post hoc test). **(E)** Flow cytometry assay of Ferrous ion (Fe^2+^) fluorescence in control or OE CBS A549 cells. **(F)** Quantified mean Fluorescence Intensity (MFI) in **(E)** (n=3, mean ± SEM, unpaired Student’s t test). **(G)** Flow cytometry analysis of lipid peroxidation through C11-BODIPY fluorescence in control or OE CBS A549 cells. FITC-BODIPY C11 and PE-BODIPY C11 represent oxidized (ox) and reduced (re) populations, respectively. **(H)** Quantification of C11-BODIPY fluorescence intensity in the specified cells in **(G)** (n=3, mean ± SEM, unpaired Student’s t test). **(I)** Western blot analysis of CBS in NCI-H23 cells transduced with lentiviral expressing shDDX3X, shDDX3X plus overexpressed (OE) CBS. β-actin was used as loading control. **(J)** Western blot analysis of DDX3X and GCLC in NCI-H23 cells transduced with lentiviral shDDX3X, shDDX3X plus overexpressed GCLC. β-actin was used as loading control. **(K)** A 6-day assessment of relative cell viability for each indicated cell line. (n=3, mean ± SEM, NS. p > 0.05, Two-way ANOVA).

**Figure S6. DDX3X regulates m^6^A modification.**

**(A)** Representative western blot analysis of DDX3X, METTL16, METTL3, METTL14, FTO, and ALKBH5 in DDX3X knockdown NCI-H23 cells. **(B and C)** Kaplan-Meier curves for **(B)** overall survival (OS) and **(C)** progression-free interval (PFI) between patients with high versus low METTL16 mRNA expression in the TCGA lung adenocarcinoma cohort. **(D)** The percentage distribution of differentially enriched m^6^A peaks according to annotation type. **(E)** Genome-wide distribution of differentially enriched m^6^A peaks in shNS vs. shDDX3X A549 cells. **(F)** mRNA metagene plots to demonstrate the distribution of m^6^A peaks within mRNA in shNS or shDDX3X A549 cells. **(G)** The motifs enriched in the MeRIP-seq peaks were analyzed by HOMER. **(H)** Scatterplots illustrating Log_2_(Fold Change) and –Log_10_(p-value) of significantly differentially enriched m^6^A peaks in shDDX3X vs. shNS A549 cells. (*p*-Value < 1E^-3^ and |Fold Change|> 1.2). **(I)** Schematic diagram of the translational reporter used to measure the activity of the 3’UTR CBS, with WT and Mut sequences inserted at the 3’ end of the firefly luciferase. **(J)** Evaluation of METTL16 protein level in NCI-H23 cells transduced with lentiviral shDDX3X as well as overexpressed WT or Mut METTL16. β-actin was employed as a loading control. **(K)** A 6-day period proliferation assay in NCI-H23 cells transduced with shNS or shDDX3X as well as overexpressed WT or Mut METTL16. (n=3, mean ± SEM, NS. p > 0.05, Two-way ANOVA).

**Figure S7. DDX3X regulates METLL16 transcription via JUND.**

**(A)**Schematic illustration of the primer design to measure the levels of detained intron-containing transcripts (middle, bottom) for the indicated genes and their total mRNA levels (top). **(B and C)** Relative levels of *METTL16* pre-mRNA **(B)** and total mRNA **(C)** in shNS or shDDX3X PC-9 cells (n=3, mean ± SEM, One-way ANOVA and Tukey's post hoc tests). **(D and E)** *JUND* and *METTL16* mRNA levels in control or siJUND PC-9 cells **(D)** and pre-mRNA levels of METTL16 **(E)** in control or siJUND PC-9 cells (n=3, mean ± SEM, One-way ANOVA and Tukey's post hoc tests). **(F)** Endogenous JUND co-immunoprecipitated with DDX3X in A549 cells. **(G)** Endogenous JUND co-immunoprecipitated with DDX3X in PC-9 cells. **(H)** Pearson correlation coefficient of DDX3X co-localization with JUND was calculated using ImageJ software. **(I)** Confocal fluorescence images of DDX3X and JUND proteins in A549 and PC-9 cells, with DDX3X in green, JUND in red, and co-localization appearing in yellow. Scale bar:10 µm.

**Figure S8. Evaluating the prognostic efficacy of DDX3X regulatory axis in LUAD.**

**(A and B)** Kaplan-Meier analysis of **(A)** overall survival (OS) and **(B)** progression-free interval (PFI) in overall patient cohorts with patients stratified into high-risk (red) and low-risk (blue) groups based on gene pairs involving DDX3X and CBS. **(C and D)** Kaplan-Meier analysis of **(C)** overall survival (OS) and **(D)** progression-free interval (PFI) in overall patient cohorts with patients stratified into high-risk (red) and low-risk (blue) groups based on gene pairs involving DDX3X and METTL16. **(E and F)** Kaplan-Meier analysis of **(E)** overall survival (OS) and **(F)** progression-free interval (PFI) in overall patient cohorts with patients stratified into high-risk (red) and low-risk (blue) groups based on gene pairs involving DDX3X and JUND. Statistical significance was determined by log-rank test.

**Figure S9. The DDX3X degrader J10 exhibits anti-tumor activity with limited side effects.**

**(A)** The synthesis of PROTAC DDX3X degraders via structure-activity optimization. **(B and C)** DDX3X protein levels in NCI-H460 **(B)** and NCI-H1975 **(C)** cells treated with increasing doses of PROTAC DDX3X degraders for 48 hours (n=3). **(D)** Representative concentration-response curves determined by CCK8 assay after treatment with different PROTAC DDX3X degraders for 48 h (n=3, mean ± SEM).

**Figure S10.** **The DDX3X degrader J10 exhibits anti-tumor activity**

**(A)** Schematic diagram of the in vivo experiment design. **(B)** Representative images of tumors in the A549 xenograft model treated with J10 (10 mg/kg) or vehicle control once every two days. **(C)** Tumor growth rate in the A549 xenograft model treated with J10 (10 mg/kg) or vehicle control once every two days. Tumor volumes were measured every 2–3 days (n=8, mean ± SEM, Two-way ANOVA). **(D)** Representative H&E staining and immunohistochemical analysis of Ki67 in the A549 xenograft model treated with J10 or vehicle control once every two days. **(E)** Quantification of Ki67 expression in **(D)** (n=8, mean ± SEM, unpaired Student’s t test). **(F)** Kaplan-Meier survival analysis showing overall survival of *Kras^G12D^;p53^fl/fl^* mice treated orally with DDX3X degrader J10 (red, n=9) versus vehicle control (blue, n=9) .Statistical significance was determined by log-rank test. **(G)** Representative H&E-stained lung sections from **(F)**. **(H)** Brightfield images of a representative patient-derived organoid treated with vehicle or J10. Images are representatives from 2 patients in each group. **(I)** Proliferation assay of patient-derived organoid treated with J10 or vehicle control as measured by ATP release assay (CellTiter-Glo 3D) (n=8, mean ± SEM, unpaired Student’s t test).

**Figure S11. Analysis of the adverse effects of the DDX3X degrader J10.**

**(A)** Immunoblot analysis of DDX3X in human bronchial epithelial BEAS-2B cells after treatment with J10, RK-33 or DMSO for 24 hours. **(B)** IC50 analysis of BEAS-2B cells treated with J10 or RK-33 for 24 hours. Data represent the mean ± SD from three biological replicates (n = 3). **(C)** The body weight of mice bearing A549 xenograft tumors treated with 10mg/kg J10 at indicated schedules (n=8, mean ± SEM, NS. p > 0.05, Two-way ANOVA with Dunnett's post hoc test). **(D)** The representative H&E staining images of mice liver tissues treated with vehicle or J10. Scale bar: 50μm. **(E-L)** Male nude mice were treated with J10 p.o. for 2 weeks. At the end of treatment, mice were sacrificed, and blood was collected for routine blood tests (n=3, mean ± SEM, NS. p > 0.05, unpaired Student’s t test).

**Figure S12. J10 showed similar capacity to induce radiosensitization as RK-33 in lung cancer cells.**

**(A)** Representative immunofluorescence images of phospho-H2AX foci in A549 cells, either untreated or pretreated with J10 or RK33, at 0, 2, 6, 12, and 24 hours post-irradiation. **(B)** Quantification of phospho-H2AX foci from **(A)**. **(C)** Representative immunofluorescence images of RAD51 foci in A549 cells, either untreated or pretreated with J10 or RK33, at 0, 6, and 24 hours post-irradiation. **(D)** Quantification of RAD51 foci from **(C)**. Data represent mean ± SEM from three independent experiments. **(E)** Quantitative comparison of LFQ intensity for DDX3X protein in A549 cells with shNS or DDX3X knockdown. **(F)** Quantitative comparison of LFQ intensity for DDX3Y protein in A549 cells with shNS or DDX3X knockdown. **(G and H)** Relative mRNA expression level of DDX3X and DDX3Y in A549 cells **(G)** and PC-9 cells **(H)** with shNS or DDX3X knockdown.

**Supplemental Tables**

**Table S1. Antibodies and reagents were used in this study.**

| **Antibody** | **Supplier** | **Catalog number** | **Dilution ratio** |
| --- | --- | --- | --- |
| Purified anti-DDX3X Antibody | Biolegend | 658602 | 1:500 |
| CBS Polyclonal antibody | Proteintech | 14787-1-AP | 1:5000 |
| GCLC Antibody | Abcam | Ab190685 | 1:20000 |
| GCLM Antibody | Abcam | Ab126704 | 1:10000 |
| METTL3 Polyclonal antibody | Proteintech | 15073-1-AP | 1:1000 |
| METTL14 Polyclonal antibody | Proteintech | 26158-1-AP | 1:1000 |
| FTO Polyclonal antibody | Abcam | Ab92821 | 1:1000 |
| ALKBH5 Polyclonal antibody | Abcam | Ab195377 | 1:1000 |
| METTL16 Antibody | Cell Signaling Technology | 17676S | 1:1000 |
| Beta Actin Monoclonal antibody | Proteintech | 66009-1-Ig | 1:5000 |
| GAPDH Monoclonal antibody | Proteintech | 60004-1-Ig | 1:10000 |
| JunD Antibody(D-9) | SantaCruz | sc-271938 | 1:250 |
| Two-Step IHC Detection Reagent | ZSGB-BIO | PV-6002 |  |
| Purified KI67(MKI67) mouse monoclonal antibody | Origene | UM870033 |  |
| Purified anti-mouse CD16/32 | Biolegend | 101302 |  |
| Goat Anti-Rabbit IgG（H+L）Secondary Antibody HRP Conjugate | Boster | BA1055 | 1:5000 |
| Goat Anti-Mouse IgG（H+L）Secondary Antibody HRP Conjugate | Boster | BA1051 | 1:5000 |
| **Reagent** | **Supplier** | **Catalog number** | |
| Lipid PeroxidationProbe-BDP581/591C11 | DOJINDO | L267 | |
| L-Cysteine hydrochloride | Med Chem Express | HY-Y0337A | |
| Erastin | Med Chem Express | HY-15763 | |
| Ferro Orange | DOJINDO | F374 | |
| Dispase | BioCoat | 354235 | |
| DNaseI | Sigma-Aldrich | DN25 | |
| ITS Liquid Media | Sigma-Aldrich | I3146 | |
| GlutaMAX™ Supplement | ThermoFisher | 35050061 | |
| HEPES(1M) | ThermoFisher | 15630080 | |
| Collagen I Rat Tail | BDBiosciences | 354236 | |
| Red Blood Cell Lysing Buffer | Sigma-Aldrich | R7757 | |
| Monarch RNA Clean up Kit | NEB | T2030L | |
| ProteinG Magnetic Beads | NEB | S1430S | |
| TransDetect Double-Luciferase Reporter Assay Kit | Transgen | FR201-01 | |
| Cycloheximide (NSC-185) | Selleck | S7418 | |
| RNA Fragmentation | Invitrogen | AM8740 | |
| EpiMark®N6-MethyladenosineEnrichmentKit | New England Biolabs | E1610S | |
| CellTiter-Glo®3DCellViabilityAssay | Promega | G9681 | |
| Matrigel | Corning | 356234 | |
| Magna RIP Kit | Millipore | 17-700 | |
| RNase Inhibitor | Novoprotein | E125 | |
| Luciferase Control RNA | Promega | L4561 | |
| CellCountingKit-8 | LIDESCI | C6030 | |
| **Plasmid** | **Supplier** | **Catalog number** | |
| pLVX-EnCMV-3×FLAG-CBS (human)-PGK-Puro | Miaoling | P32321 | |
| EX-NEG-Lv201 | GeneCopoeia | EX-NEG-Lv201 | |
| pLV201-METTL16-WT-3xflag | GeneCopoeia | CS-A3136-1 | |
| pLV201-METTL16-Mut-F187G | GeneCopoeia | CS-A3136-2 | |

**Table S2. Information of the short hairpin RNA(shRNA) and siRNA sequences in this study.**

| **Target gene** | **Sequence** |
| --- | --- |
| ShNS-shRNA control | TTCTCCGAACGTGTCACGT |
| DDX3X-shRNA | GCTGGCTCGTGATTTCTTAGA |
| DDX3X-shRNA#2 | GCAAGGATTCACTGACCTTAG |
| CBS-shRNA | CCGTCAGACCAAGTTGGCAAA |
| CBS-shRNA#2 | GACTGCGCAGAGTGGATTAAA |
| GCLC-shRNA | GCTAATGAGTCTGACCATT |
| GCLC-shRNA#2 | GTAGTATTCTGAACTACCT |
| JUND-siRNA | CCGGCAGCAUGAUGAAGAATT |
| JUND-siRNA#2 | GCCUCAUCAUCCAGUCCAATT |
| SCR-shRNA control | ACAGAAGCGATTGTTGATC |
| METTL16-shRNA | CCCTTGAGACTCAACTATATT |
| METTL16-shRNA#2 | GGAGGTGAATTAGAGTTTGTT |

**Table S3. Information of primers used for quantitative real-time PCR analyses in this study.**

| **Gene (human)** | **Sense (5’-3’)** | **Anti-sense (5’-3’)** |
| --- | --- | --- |
| *DDX3X* | ACGAGAGAGTTGGCAGTACAG | ATAAACCACGCAAGGACGAAC |
| *CBS* | GGCCAAGTGTGAGTTCTTCAA | GGCTCGATAATCGTGTCCCC |
| *CBS-3’UTR-1* | AGTCCGGAGCGCTGGGCG | GCCGTGTGCAGGGATAACGGT |
| *CBS-3’UTR-2* | CAGAGCATCCGTCTCCCCT | TGCCTGTGTTCATCCTACC |
| *CBS-3’UTR-3* | TGACTGCGCAGAGTGGATTA | CAAACACGCAAACTGCTCCC |
| *CBS-3’UTR-NC* | GTGAACAATCAGCGGCATT | GTCCAGAACAGGGAAAACC |
| *GCLC* | GGCACAAGGACGTTCTCAAGT | CAGACAGGACCAACCGGAC |
| *GCLM* | TGTCTTGGAATGCACTGTATCTC | CCCAGTAAGGCTGTAAATGCTC |
| *METTL16* | TTTCCTCGCAACAGAAGTGGA | GTCTTCTGTGGCACTTTCACC |
| *CCNE1* | GCCAGCCTTGGGACAATAATG | CTTGCACGTTGAGTTTGGGT |
| *β-actin* | CATGTACGTTGCTATCCAGGC | CTCCTTAATGTCACGCACGAT |
| *JUND* | TCATCATCCAGTCCAACGGG | TTCTGCTTGTGTAAATCCTCCAG |
| *CHD1* | GTAGCCAGTCAGGTAGCAGTG | ACTTTCGGTGGTTTTGCTTGAA |
| *MAX* | CAATCTGCGGCTGACAAACG | GCACTTGACCTCGCCTTCT |
| Nascent-*JUND* | CCTCAAACCCTGCCTTTC | CAGAATCGAACACTCTGTTCT |
| Nascent-*CBS* | AGTAAAACAGCATCGGAACACC | GCCTTCCTCTTCGCCTGA |
| Nascent-*METTL16* | GAAACGTGATTTCCCACAAA | AGTTTTCTATGGCCGTTAGG |
| Nascent-*β-actin* | AGCTCATTGTAGAAGGTGTGG | GGCATGGGTCAGAAGGATTC |
| Firefly luciferase | ATCCGGAAGCGACCAACGCC | GTCGGGAAGACCTGCCACGC |

**Table S4. Primers for genotyping the mice.**

| **Gene** | **Sense (5’-3’)** | **Anti-sense (5’-3’)** |
| --- | --- | --- |
| *Kras* | CATGGCTTGAGTAAGTCTG | GCTCCAACCACCACAA |
| *Ddx3x* | TGCCAGAAGAAAGCAGTGGATCTC | AAAGCTATCTAGTTCTGATTGTCGATACATC |
| *p53* | GCTGGAGATATGGCTTGGAGTA | AGGAGGCAGAGACAGTTGGA |

**Table S5. The sequences used for plasmid construction.**

1. **pmirGLO-CBS-3'UTR-WT:**

AGTCCGGAGCGCTGGGCGGTGCGGAGCGGGCCCGCCACCCTTGCCCACTTCTCCTTCGCTTTCCTGAGCCCTAAACACACGCGTGATTGGTAACTGCCTGGCCTGGCACCGTTATCCCTGCACACGGCACAGAGCATCCGTCTCCCCTCGTTAACACATGGCTTCCTAAATGGCCCTGTTTACGGCCTATGAGATGAAATATGTGATTTTCTCTAATGTAACTTCCTCTTAGGATGTTTCACCAAGGAAATATTGAGAGAGAAGTCGGCCAGGTAGGATGAACACAGGCAATGACTGCGCAGAGTGGATTAAAGGCAAAAGAGAGAAGAGTCCAGGAAGGGGCGGGGAGAAGCCTGGGTGGCTCAGCATCCTCCACGGGCTGCGCCGTCTGCTCGGGGCTGAGCTGGCGGGAGCAGTTTGCGTGTTTGGGTTTTTTAATTGAGATGAAATTCAAATAACCTAAAAATCAATCACTTGAAAGTGAACAATCAGCGGCATTTAGTACATCCAGAAAGTTGTGTAGGCACCACCTCTGTCACGTTCTGGAACATTCTGTCATCACCCCGTGAAGCAATCATTTCCCCTCCCGTCTTCCTCCTCCCCTGGCAACTGCTGATCGACTTTGTGTCTCTGTTGTCTAAAATAGGTTTTCCCTGTTCTGGACATTTCATATAAATGGAATCACACAA

1. **pmirGLO-CBS-3'UTR-Mut1:**

AGTCCGGAGCGCTGGGCGGTGCGGAGCGGGCCCGCCACCCTTGCCCACTTCTCCTTCGCTTTCCTGAGCCCTAAACACACGCGTGATTGGTATCTGCCTGGCCTGGCACCGTTATCCCTGCACACGGCACAGAGCATCCGTCTCCCCTCGTTAACACATGGCTTCCTAAATGGCCCTGTTTACGGCCTATGAGATGAAATATGTGATTTTCTCTAATGTAACTTCCTCTTAGGATGTTTCACCAAGGAAATATTGAGAGAGAAGTCGGCCAGGTAGGATGAACACAGGCAATGACTGCGCAGAGTGGATTAAAGGCAAAAGAGAGAAGAGTCCAGGAAGGGGCGGGGAGAAGCCTGGGTGGCTCAGCATCCTCCACGGGCTGCGCCGTCTGCTCGGGGCTGAGCTGGCGGGAGCAGTTTGCGTGTTTGGGTTTTTTAATTGAGATGAAATTCAAATAACCTAAAAATCAATCACTTGAAAGTGAACAATCAGCGGCATTTAGTACATCCAGAAAGTTGTGTAGGCACCACCTCTGTCACGTTCTGGAACATTCTGTCATCACCCCGTGAAGCAATCATTTCCCCTCCCGTCTTCCTCCTCCCCTGGCAACTGCTGATCGACTTTGTGTCTCTGTTGTCTAAAATAGGTTTTCCCTGTTCTGGACATTTCATATAAATGGAATCACACAA

1. **pmirGLO-CBS-3'UTR-Mut2:**

AGTCCGGAGCGCTGGGCGGTGCGGAGCGGGCCCGCCACCCTTGCCCACTTCTCCTTCGCTTTCCTGAGCCCTAAACACACGCGTGATTGGTAACTGCCTGGCCTGGCACCGTTATCCCTGCACACGGCTCTGTGCATCCGTCTCCCCTCGTTAACACATGGCTTCCTAAATGGCCCTGTTTACGGCCTATGAGATGAAATATGTGATTTTCTCTAATGTAACTTCCTCTTAGGATGTTTCACCAAGGAAATATTGAGAGAGAAGTCGGCCAGGTAGGATGAACACAGGCAATGACTGCGCAGAGTGGATTAAAGGCAAAAGAGAGAAGAGTCCAGGAAGGGGCGGGGAGAAGCCTGGGTGGCTCAGCATCCTCCACGGGCTGCGCCGTCTGCTCGGGGCTGAGCTGGCGGGAGCAGTTTGCGTGTTTGGGTTTTTTAATTGAGATGAAATTCAAATAACCTAAAAATCAATCACTTGAAAGTGAACAATCAGCGGCATTTAGTACATCCAGAAAGTTGTGTAGGCACCACCTCTGTCACGTTCTGGAACATTCTGTCATCACCCCGTGAAGCAATCATTTCCCCTCCCGTCTTCCTCCTCCCCTGGCAACTGCTGATCGACTTTGTGTCTCTGTTGTCTAAAATAGGTTTTCCCTGTTCTGGACATTTCATATAAATGGAATCACACAA

1. **pmirGLO-CBS-3'UTR-Mut3:**

AGTCCGGAGCGCTGGGCGGTGCGGAGCGGGCCCGCCACCCTTGCCCACTTCTCCTTCGCTTTCCTGAGCCCTAAACACACGCGTGATTGGTAACTGCCTGGCCTGGCACCGTTATCCCTGCACACGGCACAGAGCATCCGTCTCCCCTCGTTAACACATGGCTTCCTAAATGGCCCTGTTTACGGCCTATGAGATGAAATATGTGATTTTCTCTAATGTAACTTCCTCTTAGGATGTTTCACCAAGGAAATATTGAGAGAGAAGTCGGCCAGGTAGGATGAACACAGGCAATGTCTGCGCAGAGTGGATTAAAGGCAAAAGAGAGAAGAGTCCAGGAAGGGGCGGGGAGAAGCCTGGGTGGCTCAGCATCCTCCACGGGCTGCGCCGTCTGCTCGGGGCTGAGCTGGCGGGAGCAGTTTGCGTGTTTGGGTTTTTTAATTGAGATGAAATTCAAATAACCTAAAAATCAATCACTTGAAAGTGAACAATCAGCGGCATTTAGTACATCCAGAAAGTTGTGTAGGCACCACCTCTGTCACGTTCTGGAACATTCTGTCATCACCCCGTGAAGCAATCATTTCCCCTCCCGTCTTCCTCCTCCCCTGGCAACTGCTGATCGACTTTGTGTCTCTGTTGTCTAAAATAGGTTTTCCCTGTTCTGGACATTTCATATAAATGGAATCACACAA

1. **pLV201-METTL16-WT:**

ATGGCTCTGAGTAAATCAATGCATGCAAGAAATAGATACAAGGACAAACCTCCTGACTTTGCATATCTGGCATCCAAATATCCAGATTTTAAGCAGCATGTTCAGATAAATCTGAATGGAAGAGTGAGCCTTAATTTTAAAGACCCCGAAGCAGTCAGAGCTCTGACGTGTACTCTCCTAAGGGAAGATTTTGGACTTTCTATTGATATTCCATTGGAGAGACTAATTCCCACAGTTCCCTTGAGACTCAACTATATTCACTGGGTAGAAGATCTGATCGGTCACCAGGATTCTGACAAAAGTACTCTCCGAAGAGGAATTGACATAGGCACGGGGGCATCTTGCATCTACCCCTTACTTGGAGCAACCTTGAATGGCTGGTATTTCCTCGCAACAGAAGTGGATGATATGTGTTTCAACTATGCAAAGAAAAATGTGGAACAGAATAACTTATCTGATCTCATAAAAGTGGTGAAAGTGCCACAGAAGACACTCCTGATGGATGCTCTTAAAGAAGAATCTGAGATAATCTATGACTTTTGCATGTGCAACCCTCCCTTTTTTGCCAATCAATTGGAAGCCAAGGGAGTAAACTCACGAAATCCTCGAAGACCTCCGCCTAGTTCTGTTAATACAGGAGGCATCACAGAGATCATGGCAGAAGGAGGTGAATTAGAGTTTGTTAAAAGGATCATCCATGACAGTCTACAACTTAAAAAAAGATTAAGATGGTATAGCTGCATGCTGGGAAAGAAATGCAGCCTGGCGCCTCTGAAGGAGGAGCTTCGCATACAAGGGGTTCCCAAAGTAACGTACACTGAATTCTGTCAAGGTCGGACAATGAGATGGGCCTTAGCTTGGAGTTTTTATGATGATGTCACAGTACCATCACCACCAAGTAAGCGAAGAAAATTAGAGAAACCGAGAAAACCCATAACATTCGTGGTGCTGGCGTCCGTGATGAAGGAATTATCCCTCAAAGCATCACCTCTGCGCTCGGAGACGGCGGAAGGCATAGTCGTTGTCACGACATGGATTGAAAAAATTCTCACTGATTTGAAGGTCCAGCATAAACGAGTTCCCTGTGGAAAAGAGGAAGTCAGCCTTTTCCTAACGGCCATAGAAAACTCCTGGATTCATTTAAGGAGAAAGAAAAGAGAGCGTGTGAGACAGCTGAGAGAAGTTCCCCGAGCTCCTGAGGACGTCATTCAGGCCTTGGAAGAGAAAAAGCCCACCCCCAAAGAGTCTGGCAATAGCCAAGAACTGGCCAGGGGCCCCCAGGAGAGGACCCCCTGTGGGCCTGCTCTGCGGGAAGGCGAGGCTGCCGCTGTGGAGGGCCCGTGCCCGAGCCAGGAGTCCCTGTCCCAGGAGGAAAACCCGGAACCCACGGAGGATGAAAGGAGTGAGGAAAAGGGAGGGGTGGAGGTTTTGGAAAGTTGTCAAGGCTCTAGCAACGGAGCCCAGGACCAAGAGGCTTCTGAGCAGTTCGGCAGCCCAGTGGCTGAAAGGGGGAAACGTCTCCCAGGAGTGGCCGGACAGTACCTGTTTAAGTGTTTGATAAACGTTAAGAAGGAGGTGGACGATGCCTTAGTGGAGATGCACTGGGTTGAGGGCCAGAACAGGGATCTGATGAACCAGCTTTGCACCTACATACGTAACCAAATTTTCAGGCTTGTTGCAGTTAAC

1. **pLV201-METTL16-Mut-F187G:**

ATGGCTCTGAGTAAATCAATGCATGCAAGAAATAGATACAAGGACAAACCTCCTGACTTTGCATATCTGGCATCCAAATATCCAGATTTTAAGCAGCATGTTCAGATAAATCTGAATGGAAGAGTGAGCCTTAATTTTAAAGACCCCGAAGCAGTCAGAGCTCTGACGTGTACTCTCCTAAGGGAAGATTTTGGACTTTCTATTGATATTCCATTGGAGAGACTAATTCCCACAGTTCCCTTGAGACTCAACTATATTCACTGGGTAGAAGATCTGATCGGTCACCAGGATTCTGACAAAAGTACTCTCCGAAGAGGAATTGACATAGGCACGGGGGCATCTTGCATCTACCCCTTACTTGGAGCAACCTTGAATGGCTGGTATTTCCTCGCAACAGAAGTGGATGATATGTGTTTCAACTATGCAAAGAAAAATGTGGAACAGAATAACTTATCTGATCTCATAAAAGTGGTGAAAGTGCCACAGAAGACACTCCTGATGGATGCTCTTAAAGAAGAATCTGAGATAATCTATGACTTTTGCATGTGCAACCCTCCCGGTTTTGCCAATCAATTGGAAGCCAAGGGAGTAAACTCACGAAATCCTCGAAGACCTCCGCCTAGTTCTGTTAATACAGGAGGCATCACAGAGATCATGGCAGAAGGAGGTGAATTAGAGTTTGTTAAAAGGATCATCCATGACAGTCTACAACTTAAAAAAAGATTAAGATGGTATAGCTGCATGCTGGGAAAGAAATGCAGCCTGGCGCCTCTGAAGGAGGAGCTTCGCATACAAGGGGTTCCCAAAGTAACGTACACTGAATTCTGTCAAGGTCGGACAATGAGATGGGCCTTAGCTTGGAGTTTTTATGATGATGTCACAGTACCATCACCACCAAGTAAGCGAAGAAAATTAGAGAAACCGAGAAAACCCATAACATTCGTGGTGCTGGCGTCCGTGATGAAGGAATTATCCCTCAAAGCATCACCTCTGCGCTCGGAGACGGCGGAAGGCATAGTCGTTGTCACGACATGGATTGAAAAAATTCTCACTGATTTGAAGGTCCAGCATAAACGAGTTCCCTGTGGAAAAGAGGAAGTCAGCCTTTTCCTAACGGCCATAGAAAACTCCTGGATTCATTTAAGGAGAAAGAAAAGAGAGCGTGTGAGACAGCTGAGAGAAGTTCCCCGAGCTCCTGAGGACGTCATTCAGGCCTTGGAAGAGAAAAAGCCCACCCCCAAAGAGTCTGGCAATAGCCAAGAACTGGCCAGGGGCCCCCAGGAGAGGACCCCCTGTGGGCCTGCTCTGCGGGAAGGCGAGGCTGCCGCTGTGGAGGGCCCGTGCCCGAGCCAGGAGTCCCTGTCCCAGGAGGAAAACCCGGAACCCACGGAGGATGAAAGGAGTGAGGAAAAGGGAGGGGTGGAGGTTTTGGAAAGTTGTCAAGGCTCTAGCAACGGAGCCCAGGACCAAGAGGCTTCTGAGCAGTTCGGCAGCCCAGTGGCTGAAAGGGGGAAACGTCTCCCAGGAGTGGCCGGACAGTACCTGTTTAAGTGTTTGATAAACGTTAAGAAGGAGGTGGACGATGCCTTAGTGGAGATGCACTGGGTTGAGGGCCAGAACAGGGATCTGATGAACCAGCTTTGCACCTACATACGTAACCAAATTTTCAGGCTTGTTGCAGTTAAC

1. **pLVX-GCLC: NM_001498.4**

ATGGGGCTGCTGTCCCAGGGCTCGCCGCTGAGCTGGGAGGAAACCAAGCGCCATGCCGACCACGTGCGGCGGCACGGGATCCTCCAGTTCCTGCACATCTACCACGCCGTCAAGGACCGGCACAAGGACGTTCTCAAGTGGGGCGATGAGGTGGAATACATGTTGGTATCTTTTGATCATGAAAATAAAAAAGTCCGGTTGGTCCTGTCTGGGGAGAAAGTTCTTGAAACTCTGCAAGAGAAGGGGGAAAGGACAAACCCAAACCATCCTACCCTTTGGAGACCAGAGTATGGGAGTTACATGATTGAAGGGACACCAGGACAGCCCTACGGAGGAACAATGTCCGAGTTCAATACAGTTGAGGCCAACATGCGAAAACGCCGGAAGGAGGCTACTTCTATATTAGAAGAAAATCAGGCTCTTTGCACAATAACTTCATTTCCCAGATTAGGCTGTCCTGGGTTCACACTGCCCGAGGTCAAACCCAACCCAGTGGAAGGAGGAGCTTCCAAGTCCCTCTTCTTTCCAGATGAAGCAATAAACAAGCACCCTCGCTTCAGTACCTTAACAAGAAATATCCGACATAGGAGAGGAGAAAAGGTTGTCATCAATGTACCAATATTTAAGGACAAGAATACACCATCTCCATTTATAGAAACATTTACTGAGGATGATGAAGCTTCAAGGGCTTCTAAGCCGGATCATATTTACATGGATGCCATGGGATTTGGAATGGGCAATTGCTGTCTCCAGGTGACATTCCAAGCCTGCAGTATATCTGAGGCCAGATACCTTTATGATCAGTTGGCTACTATCTGTCCAATTGTTATGGCTTTGAGTGCTGCATCTCCCTTTTACCGAGGCTATGTGTCAGACATTGATTGTCGCTGGGGAGTGATTTCTGCATCTGTAGATGATAGAACTCGGGAGGAGCGAGGACTGGAGCCATTGAAGAACAATAACTATAGGATCAGTAAATCCCGATATGACTCAATAGACAGCTATTTATCTAAGTGTGGTGAGAAATATAATGACATCGACTTGACGATAGATAAAGAGATCTACGAACAGCTGTTGCAGGAAGGCATTGATCATCTCCTGGCCCAGCATGTTGCTCATCTCTTTATTAGAGACCCACTGACACTGTTTGAAGAGAAAATACACCTGGATGATGCTAATGAGTCTGACCATTTTGAGAATATTCAGTCCACAAATTGGCAGACAATGAGATTTAAGCCCCCTCCTCCAAACTCAGACATTGGATGGAGAGTAGAATTTCGACCCATGGAGGTGCAATTAACAGACTTTGAGAACTCTGCCTATGTGGTGTTTGTGGTACTGCTCACCAGAGTGATCCTTTCCTACAAATTGGATTTTCTCATTCCACTGTCAAAGGTTGATGAGAACATGAAGGTAGCACAGAAAAGAGATGCTGTCTTGCAGGGAATGTTTTATTTCAGGAAAGATATTTGCAAAGGTGGCAATGCAGTGGTGGATGGTTGTGGCAAGGCCCAGAACAGCACGGAGCTCGCTGCAGAGGAGTACACCCTCATGAGCATAGACACCATCATCAATGGGAAGGAAGGTGTGTTTCCTGGACTGATCCCAATTCTGAACTCTTACCTTGAAAACATGGAAGTGGATGTGGACACCAGATGTAGTATTCTGAACTACCTAAAGCTAATTAAGAAGAGAGCATCTGGAGAACTAATGACAGTTGCCAGATGGATGAGGGAGTTTATCGCAAACCATCCTGACTACAAGCAAGACAGTGTCATAACTGATGAAATGAATTATAGCCTTATTTTGAAGTGTAACCAAATTGCAAATGAATTATGTGAATGCCCAGAGTTACTTGGATCAGCATTTAGGAAAGTAAAATATAGTGGAAGTAAAACTGACTCATCCAAC
